# Whole-brain cFos mapping reveals the paraventricular thalamus as a key node for stress-enhanced fear learning

**DOI:** 10.3389/fnbeh.2026.1866792

**Published:** 2026-09-08

**Authors:** Michael R. Martino, Jade Baek, Megan J. Francis, Bayleigh Pagoota, Anna C. Tsyrulnikov, Jacqueline E. Paniccia, Rachel E. Clarke, Logan M. Manusky, Alexander C. W. Smith, James M. Otis

**Affiliations:** 1 Department of Neuroscience, Medical University of South Carolina, Charleston, SC, United States; 2 Ralph H. Johnson VA Medical Center, Charleston, SC, United States; 3 Hollings Cancer Center, Medical University of South Carolina, Charleston, SC, United States

**Keywords:** behavioral neuroscience, chemogenetics, post-traumatic stress disorder, stress sensitization, stress-enhanced fear learning, whole-brain cFos mapping

## Abstract

**Introduction:**

Fear learning is critical for organisms to respond appropriately to potentially harmful stimuli. In cases of post-traumatic stress disorder (PTSD), potent or persistent trauma can facilitate future aversive learning, leading to maladaptive fear responses in generally safe contexts. Despite this knowledge, the neural circuit mechanisms that underlie stress-enhanced fear learning (SEFL) remain poorly understood.

**Methods:**

We utilized brain clearing and light sheet imaging, mapping whole-brain expression of the immediate early gene cFos in mice to identify brain regions engaged by SEFL. Next, we used chemogenetics to test the causal contribution of a candidate target region, the paraventricular thalamus (PVT), to sensitized fear learning.

**Results:**

SEFL was associated with reduced activity throughout the brain, most prominently within higher order thalamic nuclei. Despite the overall reduction, cFos expression in the PVT positively correlated with a principal-component-derived predictor of subsequent sensitized fear expression. Furthermore, chemogenetic inhibition of the PVT 30 days after stress exposure prevented the acquisition of SEFL and restored freezing to control levels.

**Discussion:**

Our results provide a comprehensive whole-brain SEFL-induced cFos map. Furthermore, we reveal a novel function for one identified brain region, the PVT, in sensitized fear learning.

## 1 Introduction

Learning is a dynamic phenomenon influenced by environmental conditions and prior experience. While adapting our ability to learn relies heavily on metaplasticity, the brain’s ability to change, such adaptation can become maladaptive and even debilitating. For example, in post-traumatic stress disorder (PTSD), a traumatic event or events can cause sensitized fear learning, such that otherwise neutral stimuli can become highly fear-provoking. Indeed, sensitized fear learning is a hallmark of PTSD (Anisman, 2011; Smid et al., 2022). While we have a good understanding of the neural circuit mechanisms of standard aversive learning (Johansen et al., 2011; Nishimura et al., 2022), research focusing on sensitized fear learning has been somewhat limited.

A reliable rodent model that enables the study of sensitized fear learning is stress enhanced fear learning (SEFL) (Rau et al., 2005), in which a history of unpredictable foot shocks facilitates subsequent sensitized fear learning to a mild stressor in a novel context. Importantly, this sensitization occurs even under conditions in which the original fear memory is disrupted (Rau et al., 2005), and sensitization to future stressors persist for at least 90 days (Rau and Fanselow, 2009). Studies investigating the neural substrates involved in SEFL have identified roles for the basolateral amygdala (BLA), ventromedial prefrontal cortex (vmPFC), and dorsal hippocampus (dHPC) in regulating the acquisition and expression of stress-sensitized fear (Hersman et al., 2019; Jones et al., 2018; Pennington et al., 2017; Perusini et al., 2016). While these studies have uncovered key stress-induced neuroadaptations underlying fear hypersensitivity, a more comprehensive comparison of whole-brain activity is necessary to define the stress sensitized state and identify candidate brain regions for follow-up investigations. The thalamus is anatomically and functionally positioned to act as a key driver of long-lasting changes in fear sensitivity following traumatic stress. Indeed, there is strong evidence linking alterations in thalamic function to PTSD in human and pre-clinical studies alike (Do-Monte et al., 2015; Im et al., 2017; Magyar et al., 2025; Penzo et al., 2015; Xi et al., 2023; Yang et al., 2023; Yoshii, 2021), with the midline thalamic nuclei appearing to be of particular importance. The paraventricular nucleus of the thalamus (PVT) serves as the principal limbic output node of the periventricular midline thalamic group, receives convergent input from brainstem arousal circuits and the paraventricular hypothalamus, and projects densely to the central and basolateral amygdala and medial prefrontal cortex. These diverse afferent connections allow the PVT to integrate state relevant information with circuits encoding fear memory (Do-Monte et al., 2015; Kirouac, 2015; Penzo et al., 2015), supporting a role for the PVT in modulating fear-related behaviors. Consistent with this, PVT activity tracks motivational conflict and gates associative learning through computations that are demonstrably shaped by prior experience (Choi et al., 2019; Miranda et al., 2025; Zhu et al., 2018). Importantly, PTSD-relevant stress models alter c-Fos expression in the PVT and neighboring midline nuclei during subsequent fear-related learning, disrupting normal habituation of these regions to repeated stimuli (Della Valle et al., 2019). However, whether the midline thalamus undergoes lasting reorganization after long-term stress sensitization, and whether this reorganization is causally required for sensitized fear acquisition, has not been established.

Here, we used a 30-day SEFL paradigm combined with whole-brain cFos mapping to identify brain-wide neuroadaptations associated with stress hypersensitivity and establish a causal role for the PVT in gating sensitized fear acquisition. First, we demonstrate that mice exhibit stress enhanced fear learning in a modified SEFL paradigm, wherein mice show greater fear to a mild stressor in a novel context 30-days after initial unpredictable shock exposure. Whole-brain cFos mapping revealed robust, predominantly suppressed changes in the stress-sensitized brain, with the limbic thalamus emerging as the most affected division. Within the midline subgroup, cFos expression in the PVT and paratenial nucleus (PT) positively correlated with individual fear sensitization scores specifically in SEFL animals. Chemogenetic inhibition of the PVT selectively prevented sensitized fear acquisition, establishing midline thalamic activity as causally necessary for stress-enhanced fear encoding. Taken together, our findings describe brain wide neuroadaptations associated with SEFL and demonstrate the midline thalamic nuclei are critical for sensitized fear acquisition.

## 2 Materials and methods

### 2.1 Animals

All experiments were approved by the Institutional Animal Care and Use Committee (IACUC) at the Medical University of South Carolina in accordance with the NIH-adopted Guide for the Care and Use of Laboratory Animals. Male and female C57BL/6J mice aged 8–20 weeks were used for all experiments.

### 2.2 30 -day SEFL model

Mice were isolated at 8–12 weeks of age 4-weeks prior to Context A to allow for acclimation to their new home cages. Following 3-weeks of social isolation, animals were transported to the Mouse Behavioral Phenotyping Core (MBPC) facility and remained in their cages for 30–60 min/day and were handled for ∼1 min/day for 5 days to habituate animals to transportation and handling, respectively. Following habituation, animals were transported to the behavior core 30 min before being placed in Context A (Med Associates Inc., Product #VFC-008; cleaned with Sani-Cloth AF3 germicidal wipes [PDI, EPA Reg. No. 9480-9], lit with house lighting) for 60 min. Animals in the SEFL group received 10 pseudorandom, unsignalled foot shocks (1.0 mA, 1 sec) over 60 min, while animals in the control group spent an equivalent amount of time in Context A chambers but did not receive foot shocks. Following completion of Context A training, mice were promptly transported back to the vivarium. Twenty-four hours later mice were transported along the same route and again acclimated to the behavior core for 30 min before being placed in Context A for 8 min to test contextual fear. Mice were then left undisturbed in the vivarium for 3 weeks, followed by 5 days of acclimation to transportation and handling as performed prior to Context A using an alternate transportation route. Next, animals were habituated to Context B (Med Associates Inc., Product #VFC-008) to extinguish generalized fear. Context B was differentiated from Context A by using Sani-Cloth Plus germicidal wipes (PDI, EPA Reg. No. 9480-6), lit only with near-infrared lighting (Med Associates Inc., Product #NIR-100R2 and #NIR-022MD), and included an orange scent, A-frame, and a white-plexiglass flooring in the waste collection pan. Twenty-four hours after generalized fear was extinguished, animals were returned to Context B where they received a single tone (5 khz, 2 s) followed immediately by a single foot shock (1 mA, 2 s). Animals were removed 30 s later and returned to the vivarium. Animals were returned 24-h later to test contextual fear to Context B over 8 min. Behavioral sessions were recorded using a NIR camera and scored using VideoFreeze software (Med Associates Inc., Product #VFC-100). A subset of recordings were reviewed by a technician blinded to experimental group to validate freezing quantification.

### 2.3 Elevated plus maze (EPM)

Animals were transported to the MBPC 30 min before testing. Lighting was set to a brightness of 120 lux, and animals were placed in the center of the elevated plus maze (EPM) at the onset of behavioral testing. Animals were left in the maze for 5-min while behavior was recorded using a near infrared camera. Behavior was scored using the AnyMaze software.

### 2.4 Open field/social interaction (OF-SI)

For Open Field and Social Interaction (OF-SI) testing, animals were transported to the MBPC 30 min prior to testing. The OF-SI paradigm consisted of two phases: in phase 1, animals were placed in an open field to assess general anxiety behavior for 5 min. Following completion of phase 1, animals were briefly returned to their home cage while a novel conspecific (age and sex matched) was placed within a plexiglass barrier. Animals were returned to the open field to measure social behavior over 5 min as in phase 1. All testing was conducted with a brightness of 20–30 lux, and behavior was scored using the AnyMaze software.

### 2.5 Whole-brain clearing and cFos mapping

Brains were collected 60 min post-shock, perfused with 4% paraformaldehyde, and post-fixed overnight at 4 °C. Tissue clearing and cFos staining were performed using the AdipoClear+ protocol [adapted from (Friedmann et al., 2020)]. cFos expression was labeled using a rabbit polyclonal anti-cFos primary antibody (Abcam, ab190289; 1:1500) and an Alexa Fluor 647-conjugated donkey anti-rabbit secondary antibody (Jackson ImmunoResearch, 111-607-008; 1:800).

Samples were imaged in the horizontal orientation on a LifeCanvas Technologies SmartSPIM light-sheet fluorescence microscope (LSFM). Images were acquired using a 1.625× objective (0.2 NA) at an isotropic xyz resolution of 4 μm × 4 μm × 4 μm. A 488 nm laser was used to excite autofluorescence for registration to the Allen Brain Atlas (ABA), and a 647 nm laser was used to excite cFos immunolabeling. Brains were dehydrated in methanol and dichlromethane, then refractive index matching occurred in dibenzyl ether.

Image stacks were registered to an LSFM-optimized version of the Allen Brain Atlas Common Coordinate Framework (CCFv3) (Perens et al., 2021) using the ClearMap python package^1^ (Renier et al., 2016) on a custom in-house Linux server. Cells were detected with a peak intensity of 125–150. Cell objects were painted by a watershed until reaching this threshold, and only cells with sizes between 8 and 200 continuous pixels were included. cFos counts were corrected for regional volumes (*Cell Count / Volume*) to determine density. To account for batch effects, densities were Z-scored within each cohort (*z = (x−μ) / σ*).

Regions with a median raw signal <10 counts were excluded to prevent noise-driven artifacts. Cerebellum and olfactory regions were filtered out as these regions are a common source of technical artifacts due to damage during extraction (Pennington et al., 2024).

Regions with an effective atlas width below the spatial registration error floor of 0.10 mm were additionally excluded (Perens et al., 2021; Renier et al., 2016) (Supplementary Figure 3A). After all exclusions, 222 brain regions were included in the final analysis (see section 2.8 Statistical analysis; complete results in Supplementary Table 1). The 222 regions include both specific atlas parcels (e.g., individual thalamic nuclei) and parent-level residual nodes, which represent cells within a broader anatomical region that could not be assigned to a more specific sub-parcel at 20 μm resolution. Cell detection parameters (peak intensity threshold: 125–150; cell size bounds: 8–200 continuous pixels) were validated by iterative testing across five representational brain regions in six animals, visually comparing automated detection output to the expected cell distribution until agreement was achieved [as described in (Renier et al., 2016)]. Parameters were fixed prior to batch analysis across all samples within a given cohort. Representative images of raw cFos immunostaining, automated cell detection output, and atlas registration are provided.

### 2.6 Sensitization vector from PCA

Principal Component Analysis (PCA) was performed on a training set (30-day behavioral cohort) using three key variables: (1) Context A Freezing (Initial fear response), (2) average Habituation Freezing (Generalized fear), (3) Post-Shock Freezing (Threat reactivity). Principle component 1 (PC1) was then used to project behavioral data from the clearing cohort to calculate a standardized “Fear Sensitization” score. PC1 scores were sign-flipped where necessary to ensure that positive values consistently represented higher fear phenotypes.

### 2.7 Chemogenetic manipulation of the PVT

For PVT manipulation experiments, we performed bilateral intracranial injections of AAV2-hSyn-hM4D(Gi)-mCherry or control AAV2-hSyn-mCherry virus targeted to the PVT (Coordinates: AP: −1.55, ML: −1.13, DV: −3.30 (400 nl), −3.35 (200 nl) at a 20° angle) in male and female mice aged 8–14-weeks. Following surgery, mice were socially isolated as in the 30-day SEFL paradigm to allow for recovery and acclimation to housing conditions prior to behavioral testing. Clozapine-N-oxide (CNO; 10 mg/kg, i.p.) was administered 40 min prior to the behavioral test of interest (Context B shock or Context B test; sensitized acquisition inhibition or sensitized expression inhibition, respectively). Following experiments, viral placements were confirmed post-mortem with histology.

### 2.8 Statistical analysis

All analyses were conducted in R (version 4.5). Freezing behavior was analyzed via two-way ANOVA (Group × Sex) followed by Tukey’s HSD *post-hoc* tests. For longitudinal habituation experiments, changes in freezing over days were analyzed using a repeated measures ANOVA (or Linear Mixed Model) with Day as a within-subjects factor and Group as a between-subjects factor. For animals receiving PVT inhibition during sensitized acquisition, a two-way ANOVA (Stress × Virus) was used. For animals receiving PVT inhibition during sensitized expression testing, unpaired two-tailed *t*-test (SEFL-mCherry vs. SEFL-Gi) was used. Due to unbalanced group sizes in the exploratory cohorts used for EPM and OF-SI tests, data were collapsed across sex and analyzed using a one-way ANOVA (or two-tailed *t*-test) to assess the main effect of Stress Group.

Brain-wide c-Fos density was analyzed using a hierarchical negative binomial generalized linear model (NB-GLM) framework. NB-GLM was selected to account for overdispersion common in cFos count data (Love et al., 2014; Robinson et al., 2010). At the individual region level, a separate NB-GLM was fit for each of 222 regions with the formula: *signal*∼*Group* + *Cohort* + *offset*(*log*⁡(*Volume*)), where signal is the raw c-Fos+ cell count, Group is the SEFL/Group factor, Cohort controls for batch effects across tissue processing runs, and offset(log(Volume)) normalizes for region size. Effect sizes are reported as log rate ratios ($L\,o\,g\,R\,R=\log\left[\frac{S\,E\,F\,L\,c\,F\,o\,s\,d\,e\,n\,s\,i\,t\,y}{C\,o\,n\,t\,r\,o\,l\,c\,F\,o\,s\,d\,e\,n\,s\,i\,t\,y}\right],a\,d\,j\,u\,s\,t\,e\,d\,f\,o\,r\,C\,o\,h\,o\,r\,t$); rate ratios (exp[*LogRR*]) reflect the change in SEFL relative to controls. Profile likelihood 95% confidence intervals were computed for each regional LogRR. Multiple comparisons across all 222 regional tests were corrected using the Benjamini-Hochberg (BH) false discovery rate procedure [*q* < 0.05 threshold; (Benjamini and Hochberg, 1995)]. To assess whether any major brain division was disproportionately represented among significant hits, the number of regions reaching nominal (*p* < 0.05) and FDR-corrected (*q* < 0.05) significance was summed for each of the Allen CCFv3 divisions present in our data. The division with the greatest number of affected regions was then analyzed using the same NB-GLM applied to each of the 7 CCFv3 functional thalamic subgroups, with BH-FDR correction applied separately to these regions. Thalamic subgroups were defined following CCFv3 functional ontology (Vertes et al., 2022; Wang et al., 2020). Complete results for all 222 regions are provided in Supplementary Table 1. NB-GLM analysis used R (v 4.5.3) with the MASS package (v 7.3).

## 3 Results

### 3.1 Previous unpredictable foot shock experience sensitizes mice to fear learning 30-days later

**FIGURE 1:**
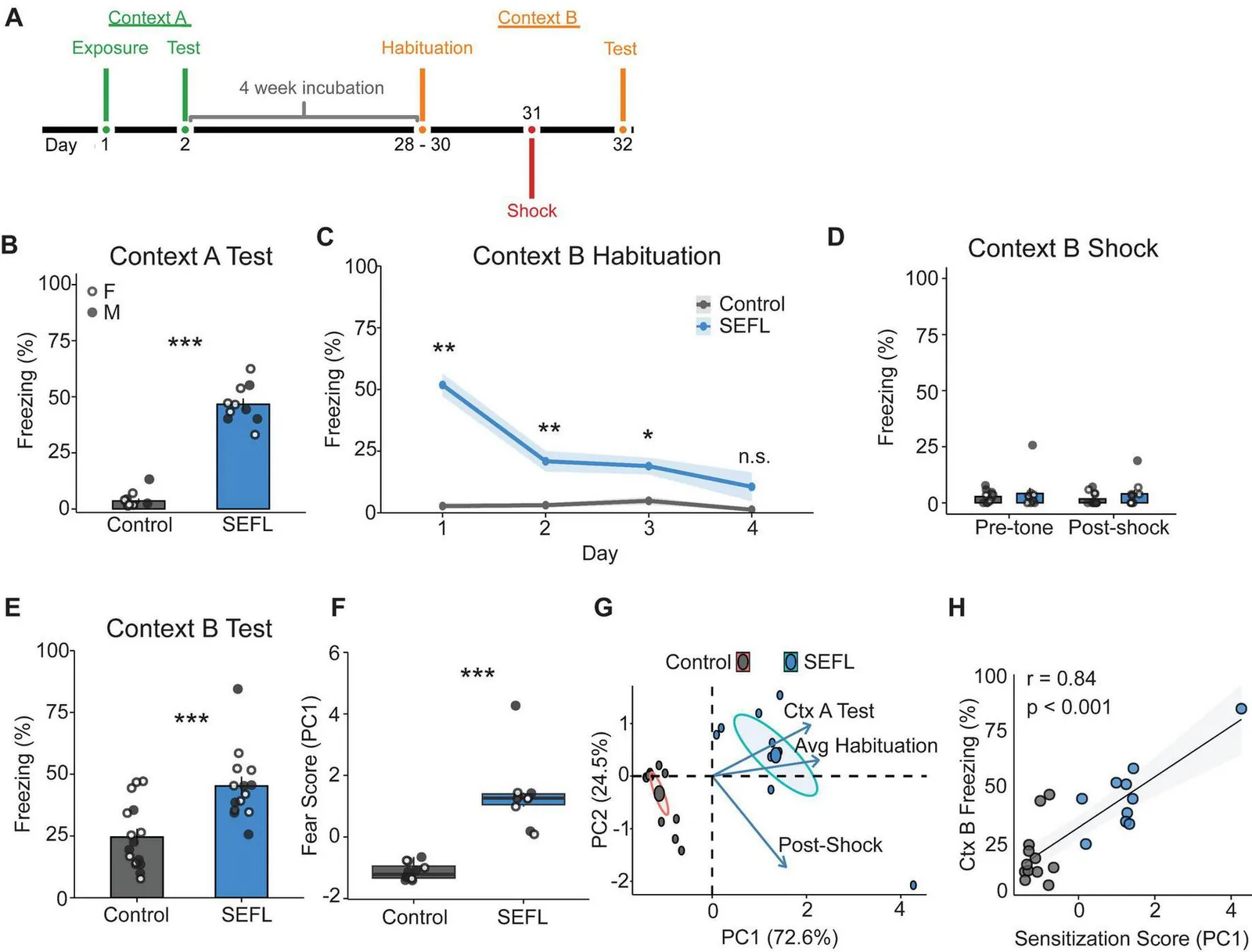
30-day SEFL model. **(A)** Experimental timeline for 30-day SEFL behavioral paradigm. **(B)** Freezing behavior during Context A test in control and SEFL animals. **(C)** Freezing behavior throughout Context B habituation trials in control and SEFL animals. **(D)** Freezing behavior during the pre-tone and post-shock periods of Context B single shock fear conditioning in control and SEFL mice. **(E)** Freezing behavior during Context B test in control and SEFL mice. **(F)** Principle component (PC) 1 scores of 30-day animals. **(G)** PCA loading biplot of variables used in 30-day behavior PCA to discover sensitization vector (PC1). **(H)** Correlation between PC1 and Context B freezing behavior in 30-day animals. *p < 0.05, **p < 0.01, ***p < 0.001.

Previous studies have shown that a single sufficiently stressful experience sensitizes rodents to future fear learning, leading to significantly greater contextual and cued fear learning in sensitized animals compared to stress naïve controls, and that this effect persists for at least 90 days in rats (Rau and Fanselow, 2009; Rau et al., 2005). To better understand the long-term consequences of traumatic stress, we used a 30-day model of stress enhanced fear learning (SEFL; Figure 1A). Animals were randomly assigned to SEFL or control conditions prior to Context A conditioning. In Context A, SEFL animals received 10 unsignalled foot shocks (1 s, 1 mA) over 60-min, while control animals spent an equivalent amount of time with no foot shocks. Twenty-four hours later animals were returned to Context A and contextual fear was assessed. As expected, SEFL animals displayed significantly higher freezing to the context than control animals (Figure 1B). No effects of sex (*p* = 0.83) or group × sex interaction (*p* = 0.51) were detected.

A PTSD diagnosis requires symptoms persist for at least 4-weeks following the traumatic experience, differentiating it from acute stress disorder (DSM-V). Importantly, the neural representations of the experience undergo significant changes through the process of systems consolidation during this time, in which the original memory becomes distributed throughout the brain (cortex) beyond sub-cortical areas like the hippocampal (HPC) and amygdalar (AMG) nuclei that are critical for acute memory (DeNardo et al., 2019; Sacco and Sacchetti, 2010). Indeed, prefrontal ensembles continue to undergo changes for at least 4-weeks following conditioning (DeNardo et al., 2019). Similarly, secondary sensory areas contribute to fear retrieval 1 month, but not 1 day, after fear conditioning (Sacco and Sacchetti, 2010). Considering these findings, we allowed animals to remain undisturbed for 4-weeks following Context A conditioning. Prior to Context B fear conditioning, SEFL animals exhibited fear generalization despite differences in transportation and key characteristics of the fear chambers (Figure 1C) which was successfully extinguished over 4-days of context habituation (Figure 1C). Following extinction of generalized fear, we next subjected all animals to a single foot shock (2 s, 1 mA) following a brief auditory cue (5 khz, 90 db, 2 s), with no differences observed between groups during the pre-tone and post-shock periods (Figure 1D). When returned to Context B 24-h later, stress-sensitized animals demonstrated significantly greater contextual freezing (Figure 1E) in line with previously published reports (Gonzalez et al., 2021; Hersman et al., 2019; Nishimura et al., 2026; Pennington et al., 2017; Perusini et al., 2016; Rau and Fanselow, 2009; Rau et al., 2005). No significant main effects of sex (*p* = 0.40) or group × sex interactions (*p* = 0.42) were detected at recall.

We next performed principal component analysis (PCA) across behavior sessions to discover a sensitization vector that best captured the observed behavioral differences between groups (Figures 1F, G). The sensitization vector captured 72.6% of the variance and was significantly different between groups (Figure 1F). This vector had a strong and significant correlation with Context B freezing behavior (Figure 1H) that better predicted fear sensitized freezing behavior than any individual behavior.

### 3.2 Stress sensitization produces a generalized disruption of safety-seeking strategies

To determine if our 30-day SEFL model resulted in disruptions of other relevant behaviors beyond context-specific fear sensitization, we performed elevated plus maze (EPM) and open-field (OF) testing in our animals (Supplementary Figure 1A) following Context B conditioning and recall. In the EPM, SEFL animals spent significantly more time spent in the open arms (Supplementary Figure 1B) and significantly less time in the closed arms (Supplementary Figure 1C). This shift was accompanied by fewer entries into the closed arms (Supplementary Figure 1D), though overall locomotion on the maze was not statistically different between groups (*p* = 0.22). Twenty-four hours later, mice demonstrated a similar disruption of ethological defensive behavior in the OF test. SEFL animals spent significantly less time in the safe corners of the arena (Supplementary Figure 1E) and exhibited reduced overall locomotor activity (Supplementary Figure 1F).

Notably, while defensive behaviors were disrupted, social behaviors remained intact. Following the first phase of the OF test where animals were allowed to freely explore the arena, a novel conspecific social target was added to the maze (Supplementary Figure 1A). During the social interaction phase, no significant differences were observed between groups in social motivation (*p* = 0.84), social zone entries (*p* = 0.83), or social locomotion (*p* = 0.33). We posit that entry to the closed arms of the EPM or the corners of the OF may activate a generalized fear of the enclosed fear chambers mice received foot shocks in, which disrupts ethologically conserved defensive strategies to reflect the changing environment.

### 3.3 Traumatic stress alters brain wide patterns of activation during acute stress

**FIGURE 2:**
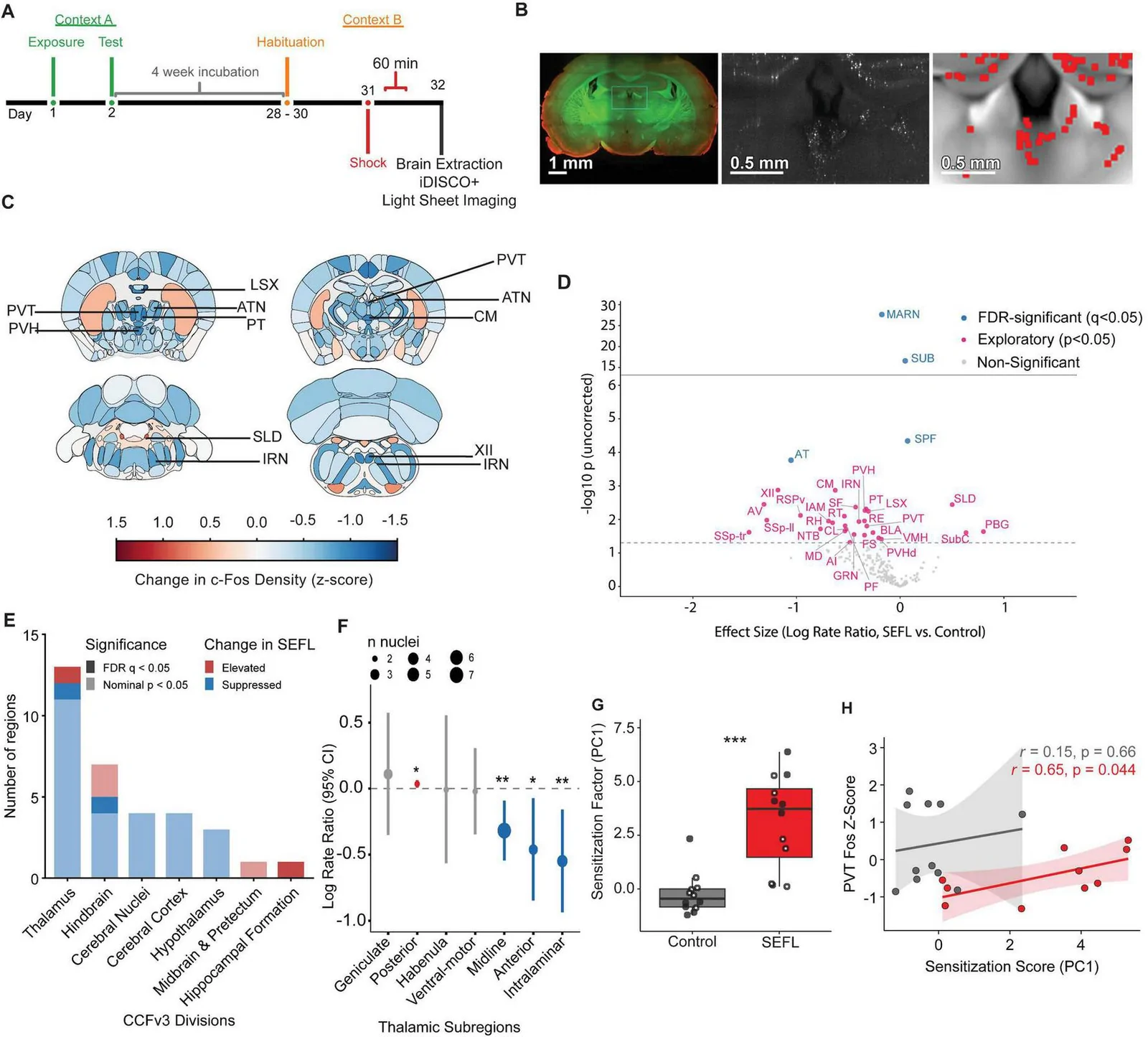
Whole brain cFos mapping of the stress sensitized brain. **(A)** Experimental timeline for whole brain cFos mapping of the stress sensitized brain. **(B)** Representative coronal images from whole-brain imaging and cell registration. From left to right: max intensity projection of autofluorescence and cFos channels, max intensity projection taken from a section containing the midline thalamus, and cFos+ points registered to the brain atlas. **(C)** Representative mean difference z-score heatmaps throughout the mouse brain. **(D)** Volcano plot of NB-GLM -log_10_ *p*-values and effect size. Nominal regions are shown in pink, while regions that survive FDR are shown in blue. **(E)** Division counts of regions identified in panel **(D)**. **(F)** 95% CI of effect sizes from NB-GLM of cFos density in thalamic subregions. **(G)** Stress sensitization component scores for control and SEFL animals. **(H)** Correlations between z-score normalized cFos counts in the PVT and stress sensitization scores in control (gray) and SEFL (red) mice. *p < 0.05, **p < 0.01, ***p < 0.001.

After establishing the relevance of our 30-day SEFL model, we next aimed to define the whole-brain signature of stress sensitization during acute fear. Using the previously described 30-day SEFL paradigm, brains were collected from mice 60 min after foot shock in Context B to perform whole-brain cFos mapping (Figure 2). Brains were optically cleared (iDISCO+) and stained for cFos, then imaged via light-sheet microscopy and mapped to the Allen Brain Atlas Common Coordinate Framework (CCFv3) for automated segmentation and quantification (Figure 2B). Full behavioral data for this cohort are provided in Supplementary Figure 2.

To identify regions differing between groups, we applied a hierarchical NB-GLM framework across 222 brain regions with BH-FDR correction (see section “2.8 Statistical analysis”; Supplementary Table 1). Four regions survived FDR correction (*q* < 0.05; Figure 2D): MARN (Magnocellular reticular nucleus), SUB (Subiculum), SPF (Subparafascicular nucleus), and AT (Anterior thalamic region). An additional 29 regions reached uncorrected *p* < 0.05 (Figure 2D and Supplementary Table 1).

Of the 33 regions reaching nominal significance (*p* < 0.05), the thalamus contained nearly twice as many (13) identified regions as the next most represented division (7), and 2 of the 4 FDR-significant regions were also found in the thalamus (Figure 2E). Given the diverse functions associated with distinct thalamic subregions (Steele et al., 2026; Taber et al., 2004; Vertes et al., 2022; Yang et al., 2023; Yoshii, 2021), we performed follow up targeted analysis of c-Fos density throughout the following thalamic subregions defined by the CCFv3: Geniculate, Posterior, Habenula, Ventral-motor, Midline, Anterior, and Intralaminar. Following BH-FDR correction, three subgroups showed significantly reduced cFos in SEFL animals (Figure 2F): Midline (LogRR = -0.32, q ≈ 0.021), Intralaminar (LogRR = -0.55, q ≈ 0.021), and Anterior (LogRR = -0.46, q ≈ 0.033). The Anterior subgroup (AD, AV, IAD) comprises the anterior nuclear group of the Papez circuit, involved in hippocampal-prefrontal communication and contextual memory. In addition, the posterior subgroup showed a marginal positive increase in cFos (Figure 2F). Given the small increase (<4%) this subgroup as not considered in subsequent analyses. No significant effects were detected in sensory relay subgroups (Geniculate, Ventral-motor; *p* > 0.64), indicating that SEFL selectively suppresses limbic and arousal-linked thalamic nuclei rather than primary sensory relay. Within the Midline subgroup, the paraventricular thalamus (PVT) and paratenial nucleus (PT) had nominal significance in the whole-brain screen but did not survive FDR.

To determine which candidate regions were functionally relevant to the behavioral phenotype in SEFL animals, we calculated the sensitization vector for this cohort using the PCA approach described in Figure 1. Consistent with earlier findings, SEFL animals displayed significantly greater sensitization scores than controls (Figure 2G). We then correlated cFos expression across candidate regions with each animal’s sensitization score (Supplementary Figure 5). This revealed a group-specific divergence: in SEFL animals, cFos expression in individual midline thalamic nuclei (PVT and PT) showed a significant positive correlation with the sensitization vector, a relationship that was absent in controls (Figure 2H and Supplementary Figure 5). The PVT is a midline thalamic structure that receives inputs from brainstem and hypothalamic stress centers (including the IRN and PVH), and projects to the amygdala and prefrontal cortex to gate fear responses (Do-Monte et al., 2015; Penzo et al., 2015). Furthermore, recent work suggests the PVT tracks situational salience during high-conflict or high-salience events (Zhu et al., 2018), and its functional role appears to be modified by past experiences (Choi et al., 2019). Taken together, our findings strongly suggest the PVT is functionally involved in sensitized fear acquisition.

### 3.4 PVT activity is necessary for stress sensitized fear acquisition

**FIGURE 3:**
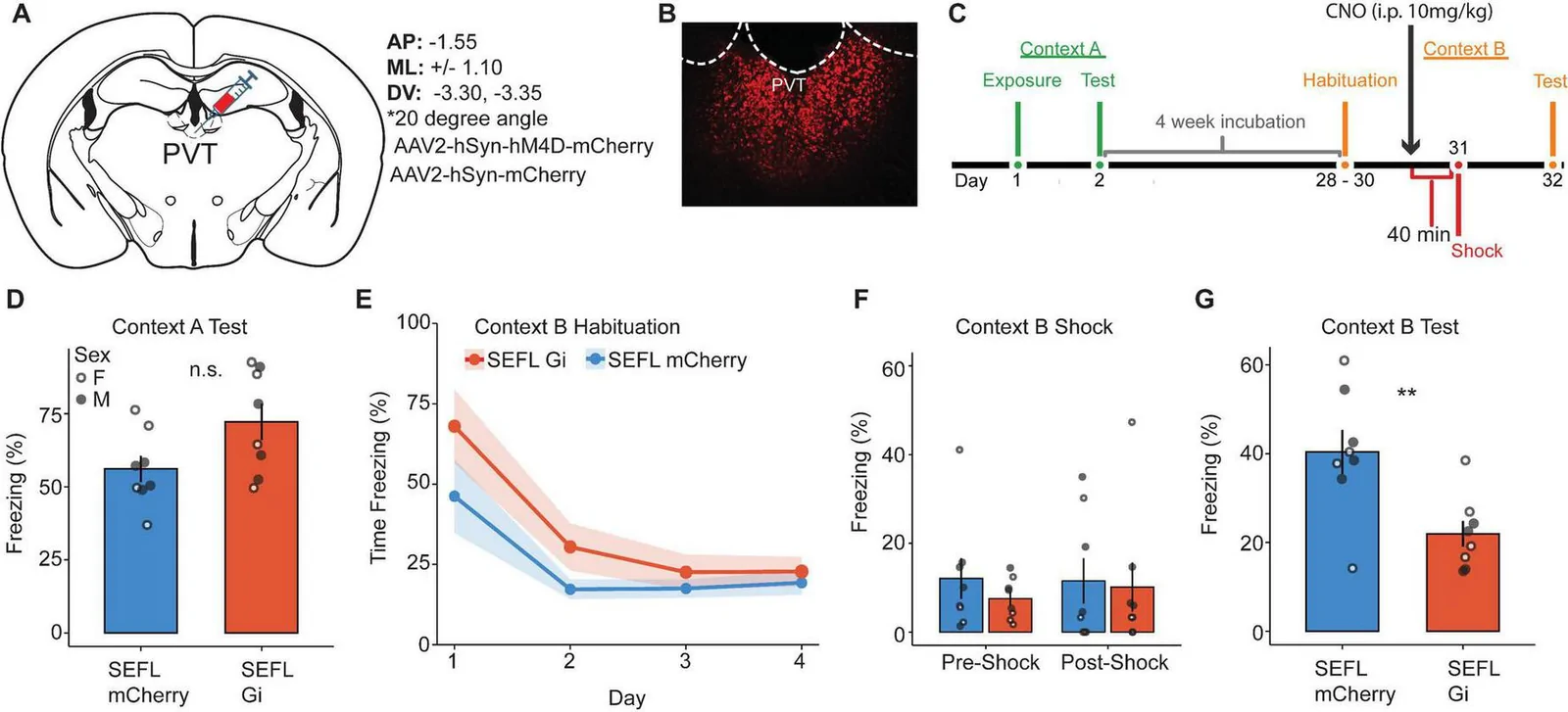
Chemogenetic inhibition of the PVT prevents stress sensitized fear learning. **(A)** Viral targeting of midline thalamus with AAV2-hM4D-mCherry (SEFL Gi) or AAV2-mCherry (SEFL mCherry) virus. **(B)** Representative image of viral expression in the PVT within the midline thalamus. **(C)** Experimental timeline for chemogenetic inhibition of the PVT during sensitized fear acquisition. **(D)** Freezing behavior during Context A test. **(E)** Freezing throughout Context B habituation trials. **(F)** Freezing behavior during stress sensitized fear acquisition during chemogenetic inhibition of the PVT. **(G)** Freezing behavior during sensitized fear retrieval. **p < 0.01.

Despite the PVT’s known involvement in fear and experience-dependent learning, its causal role in remote fear sensitization remains unknown. Based on our current findings, we hypothesized that in SEFL animals, the PVT acts as a maladaptive gain control mechanism during memory acquisition, resulting in an exaggerated fear response. To test this hypothesis, we performed targeted chemogenetic inhibition of the PVT during sensitized fear acquisition (Figure 3).

Mice received intracranial injections of Gi-DREADD or a control mCherry virus targeting the PVT 4-weeks before Context A conditioning. Virus expression did not affect initial fear expression during testing in Context A (Figure 3D). Although 4-days was sufficient to successfully extinguish fear in 30-day SEFL animals, individual time courses to extinction were heterogenous. To ensure a standardized low-fear baseline, we utilized a criterion-based habituation where animals underwent habituation to Context B until freezing levels reached <15%. All groups successfully learned to extinguish generalized fear (Figure 3E). Following successful extinction of generalized fear, we treated mice with 10 mg/kg of CNO (i.p.) 40 min before a single tone-shock pairing in Context B. Mice displayed no differences in freezing behavior during fear acquisition (Figure 3F), indicating that PVT inhibition did not alter acute shock reactivity.

When returned to Context B 24-h later to test fear expression, prior inhibition of the PVT successfully blocked the stress-sensitized fear expression (Figure 3G). Specifically, while SEFL-mCherry animals exhibited robust freezing to Context B, SEFL-Gi animals showed significantly reduced freezing behavior. These data indicate PVT inhibition during Context B shock effectively rescues the phenotype to levels observed in control animals from our first set of experiments (Figure 1). To test if the PVT would function similarly to control standard fear acquisition in stress naïve animals, we performed the same PVT chemogenetic inhibition during context B shock in control mice and found no change in freezing behavior during context B fear retrieval (Supplementary Figure 6F). Histological verification of viral placements, including sensitivity analysis of expression spread and subsequent behavior, is provided in Supplementary Figure 8. These results confirm that PVT activity during the acute, single shock is necessary for the acquisition of the sensitized fear memory, but not standard fear encoding in stress naïve animals.

### 3.5 PVT activity is not required for the expression of sensitized fear

To determine if the PVT mediates the expression of the sensitized state independent from acquisition, we performed a second experiment in an independent cohort of SEFL animals. SEFL mice were injected with Gi-DREADD or mCherry virus in the PVT and underwent the standard 30-day SEFL protocol. Since the extended habituation did not significantly predict lower fear recall in the previous set of experiments, animals were habituated to Context B for 3 days, at which point freezing levels remained similar between groups (Supplementary Figure 7D). Twenty-four hours after the Context B shock, mice received 10 mg/kg CNO (i.p.) 40 min prior to the Context B recall test. Inhibition of the PVT during testing had no effect on fear expression as both SEFL-mCherry and SEFL-Gi animals displayed similarly high levels of freezing (Supplementary Figure 7F).

Taken together, these findings show that the PVT is necessary for sensitized fear acquisition long after the original traumatic event. Importantly, PVT chemogenetic inhibition reduced fear memory recall to levels observed in control animals, specifically preventing the escalation of the fear memory while leaving the sensory representation intact. These data indicate that the PVT acts as a history-dependent gain control mechanism that amplifies fear encoding specifically in traumatized brains.

## 4 Discussion

Using a 30-day model of stress sensitization, we demonstrate that sensitized animals exhibit altered patterns of cFos activation throughout the brain during fear acquisition in Context B. At the whole-brain level, FDR-corrected differences were observed in four regions: suppression in the MARN and AT, and elevations in the SUB and SPF. The thalamus was the most overrepresented division among regions reaching significance. Follow up analysis identified significantly reduced cFos in the midline and intralaminar thalamic subgroups. Within the midline thalamic nuclei, the PVT showed nominally reduced cFos and positively correlated with PCA derived fear sensitization scores in SEFL, but not control, animals. Targeted chemogenetic inhibition of the PVT during subsequent fear acquisition prevented the development of sensitized fear without impairing standard fear acquisition. Conversely, inhibition during memory retrieval had no effect on the expression of sensitized fear. Together, these findings indicate that the PVT drives the acquisition, rather than the expression, of the stress-sensitized fear memory.

### 4.1 Brain-wide cFos map of stress sensitized fear learning

Our whole brain cFos mapping experiment identified changes in expression at regional and subregion atlas levels. After adjusting for multiple comparisons, our analyses found significantly lower cFos in the MARN, AT, intralaminar thalamic nuclei, and midline thalamic nuclei, with significantly greater cFos in the SUB and SPF. This parallels findings showing reductions in IEG gene expression following chronic stress (Xiong et al., 2026) and may be reflective of increased specificity of ensembles that exert greater control of behavior despite reductions in size (Josselyn and Tonegawa, 2020; Thibeault et al., 2025). This could amplify learning through pre-existing engrams present in the SEFL but not control animals, reducing signal to noise and strengthening the encoding of the emotional memory. In addition, novelty has been shown to affect cFos activation throughout the brain (Bourgeois et al., 2012; Kabbaj and Akil, 2001; Montag-Sallaz et al.,1999), potentially contributing to differences between groups. Interestingly, the thalamus contained the most significantly different regions, primarily in higher-order nuclei. Whereas primary thalamic nuclei relay sensory information, higher-order regions are characterized by cortico-thalamo-cortical loops (Sherman and Guillery, 2013) and are implicated in integrative as well as other higher order cognitive processes (Halassa and Kastner, 2017). The anterior thalamic subgroup (AD, AV, IAD) – nuclei of the Papez circuit that mediate hippocampal-prefrontal-thalamic communication – also showed significant suppression, consistent with a broader disruption of experience-dependent memory circuits in the stress-sensitized state. Thus, we predicted that the overall trend in reduced thalamic c-Fos was driven primarily by higher-order thalamic nuclei.

### 4.2 Stress sensitized animals display altered thalamic cFos expression patterns

While the thalamus has historically been viewed as a relay for subcortical information to the cortex, accumulating evidence demonstrates that thalamic nuclei are functionally heterogeneous: a minority support the canonical sensory relay function while the majority participate in higher-order integrative and limbic processes (Halassa and Kastner, 2017; Vertes et al., 2022). To better characterize the functionally relevant areas, we grouped thalamic nuclei according to seven CCFv3 functional subgroups (Wang et al., 2020). Following BH-FDR correction, we identified significant suppression in the Midline, Intralaminar, and Anterior subgroups, collectively referred to as the limbic thalamus, characterized by direct projections to the amygdala, prefrontal cortex, and hippocampus rather than primary sensory cortices (Kirouac, 2015; Vertes et al., 2022). This selectivity indicates that SEFL reorganizes limbic-thalamic communication specifically, rather than producing a global suppression of thalamic activity.

This pattern is consistent with prior evidence that stress models alter midline thalamic IEG expression during fear-related learning. Single prolonged stress disrupts normal habituation of c-Fos specifically in the PVT, nucleus reuniens, and medial habenula during subsequent fear extinction training (Della Valle et al., 2019). Our findings extend this observation from an acute stress model to a 30-day sensitization paradigm, suggesting that midline thalamic reorganization is a persistent feature of the stress-sensitized state rather than an acute response to the stressor itself. This interpretation is consistent with structural neuroimaging in human PTSD, where disrupted intrathalamic and thalamocortical covariance networks have been identified, with aberrant coupling specifically involving limbic thalamic nuclei (Steele et al., 2026). Together, these findings suggest that altered midline thalamic connectivity is a conserved neurobiological feature across rodent stress models and human PTSD.

Experience-dependent plasticity may explain how prior stress reconfigures thalamic computations for future learning. Prior rewards experience has been shown to recruit novel GABAergic populations in the lateral hypothalamus during subsequent aversive learning, populations absent in rewards-naïve animals (Sharpe et al., 2021), demonstrating that experience can fundamentally alter which neural populations are engaged during future associative encoding. Counter-conditioning experiments similarly show that the behavioral consequence of PVT manipulations depends on prior conditioning history (Choi et al., 2019). Within this framework, the 30-day incubation period may allow the midline thalamus to undergo analogous experience-dependent reconfiguration, establishing a new computational baseline that biases subsequent fear encoding. Within the midline subgroup, cFos in the PVT and paratenial nucleus (PT) showed strong positive correlations with the individual fear sensitization score specifically in SEFL animals, pointing to these nuclei as the most behaviorally relevant nodes within the broader limbic thalamic reorganization. Thus, we predicted that the identified adaptations in thalamic cFos expression may have functional consequences for behavior.

### 4.3 The PVT’s role in threat computations and sensitization

The PVT occupies a critical interface between stress-state signaling and fear memory circuits. It receives convergent input from brainstem arousal centers and the paraventricular hypothalamus and projects primarily to the central and basolateral amygdala and medial prefrontal cortex, the canonical circuit for fear acquisition and regulation (Do-Monte et al., 2015; Kirouac, 2015; Penzo et al., 2015). Importantly, PVT computations are shaped by motivational history rather than current inputs alone (Choi et al., 2019; Miranda et al., 2025), making it well positioned to act as a history-dependent gain-control node that amplifies fear encoding specifically when the organism arrives at conditioning in a stress-sensitized state. Consistent with this, despite an overall reduction in cFos expression in SEFL animals, residual PVT activation strongly correlated with individual fear sensitization scores, and chemogenetic inhibition of the PVT selectively prevented sensitized fear acquisition without impairing standard fear learning in stress-naïve controls. Indeed, chronic or repeated stress consistently reduces IEG expression in stress-relevant circuits (Xiong et al., 2026), which may reflect homeostatic remodeling rather than functional silencing. For example, repeated engagement in a task has been shown to reduce engram size yet simultaneously increase the behavioral effect of manipulation (Thibeault et al., 2025).

Though viral expression was observed throughout the midline thalamus, existing literature suggests such a change may be primarily orchestrated by the PVT. A recent report investigating stress-enhanced fear responding (SEFR) found that while the posterior PVT mediates the expression of non-associative sensitization to loud tones following acute stress, it is not required for the acquisition of the SEFL phenotype (enhanced contextual fear) (Nishimura et al., 2026). We posit that the divergence in our findings is likely driven by critical differences in stress severity, incubation time, and paradigm design. First, our SEFL model included a highly salient stressor (10 shocks) followed by a 30-day incubation period, which likely drives structural network reorganization not captured in acute, mild-stress models (4 shocks, 8 days). Indeed, previous studies have found the PVT→CeA circuit involved in fear expression is necessary for remote, but not acute, fear maintenance and expression (Do-Monte et al., 2015). In addition, animals in the acute SEFR study were tested in a different context, presenting an absolute, conflict-free threat. In contrast, our SEFL animals were extensively habituated to Context B prior to receiving a foot shock. As recent literature indicates the PVT is required for computing relative threat (Miranda et al., 2025), the necessity of the PVT in our acquisition model likely reflects the need to update conflicting information about the relative threat of the chamber, a conflict absent in stress naïve animals. Finally, our inclusion of a brief, 2-s pre-shock tone may have acted as a discrete salience cue, further recruiting the PVT to drive learning (Zhu et al., 2018). These important considerations warrant follow up investigations to better understand if these differences map to distinct ethological constructs of PTSD in clinical populations.

### 4.4 Limitations and conclusions

While our findings establish the PVT as a critical node in stress-sensitized fear acquisition, several methodological limitations warrant consideration. Because cFos expression and systemic DREADD manipulations lack temporal precision, the exact window of PVT involvement remains undefined. Specifically, it is unclear whether the PVT drives this maladaptive learning via real-time threat computation during the shock exposure itself, or during the immediate post-conditioning consolidation window. Future studies utilizing *in vivo* imaging and optogenetics will be essential to map these precise temporal circuit dynamics. Although post-mortem assessment of viral expression was observed primarily in the PVT, we identified variable spread to adjacent midline nuclei in a subset of control Gi and mCherry animals (Supplementary Figure 8). No differences were observed in freezing behavior of control animals regardless of the extent of viral spread. Future experiments can better test the role of individual thalamic nuclei in fear sensitization using more precise viral targeting via localized delivery of DREADD ligands to the PVT or targeted optogenetic manipulations. Furthermore, future behavioral models could employ Context A extinction to definitively isolate the stress-sensitized state from stimulus-specific fear generalization. Beyond identifying a novel role for the PVT in remote stress sensitization, this work provides the first comprehensive, whole-brain functional dataset of remote stress sensitization, offering a valuable dataset for investigating the broader neural networks driving maladaptive fear. Our findings provide a novel framework for understanding how prior trauma remodels brain region function, establishing the PVT as a promising circuit target for interventions targeting sensitized fear learning.

## Data Availability

The datasets presented in this study can be found in online repositories. The names of the repository/repositories and accession number(s) can be found below: https://github.com/Otis-Lab-MUSC/martino-et-al-fbn-2026.

## Footnotes

1
www.github.com/alexcwsmith/tree/python3
