# Supplementary material for: Whole-brain cFos mapping reveals the paraventricular thalamus as a key node for stress-enhanced fear learning
Source: Front Behav Neurosci. 2026 Sep 8;20:1866792. doi: 10.3389/fnbeh.2026.1866792 (PMC13593807; doi:10.3389/fnbeh.2026.1866792)
Supplement: Supplementary file 1 [file Data_Sheet_1.pdf]

Supplementary Material

1.1 Supplementary Figures

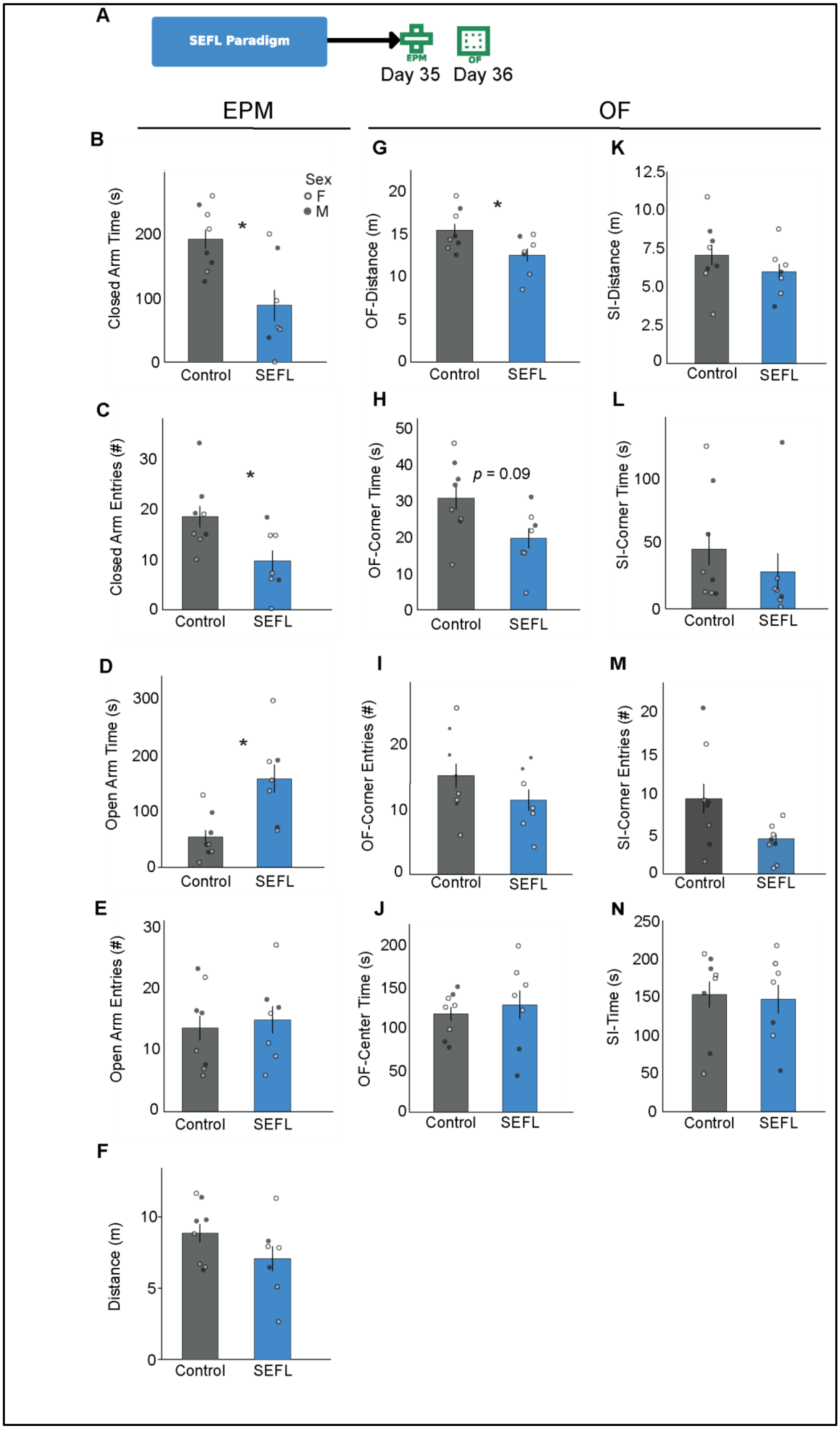
A
SEFL Paradigm
EPM
OF
Day 35
Day 36
EPM
OF
B
Sex
F
M
Closed Arm Time (s)
Control
SEFL
C
Closed Arm Entries (#)
D
Open Arm Time (s)
E
Open Arm Entries (#)
F
Distance (m)
G
OF-Distance (m)
H
OF-Corner Time (s)
p = 0.09
I
OF-Corner Entries (#)
J
OF-Center Time (s)
K
SI-Distance (m)
L
SI-Corner Time (s)
M
SI-Corner Entries (#)
N
SI-Time (s)

Supplementary Figure 1. EPM and OF Behavior of 30-day SEFL Mice (A) Experimental timeline for EPM and OF testing. (B – F) EPM behavior. (B) Time spent in the closed arms. (C) Number of entries into closed arms. (D) Time spent in the open arm. (E) Number of entries into open arms. (F) Locomotor activity. (G-J) Behavior during an open-field test. (G) Locomotor activity during OF test. (H) Time spent in the corners of the box. (I) Total number of corner entries. (K-N) Behavior during an open-field test with a novel conspecific social target (SI). (K) Locomotor activity during OF-SI test. (L) Time spent in the corners. (M) Number of entries to the corners. (N) Time spent interacting with social target. * = $p \leq 0.05$, ** = $p \leq 0.01$, *** = $p \leq 0.001$

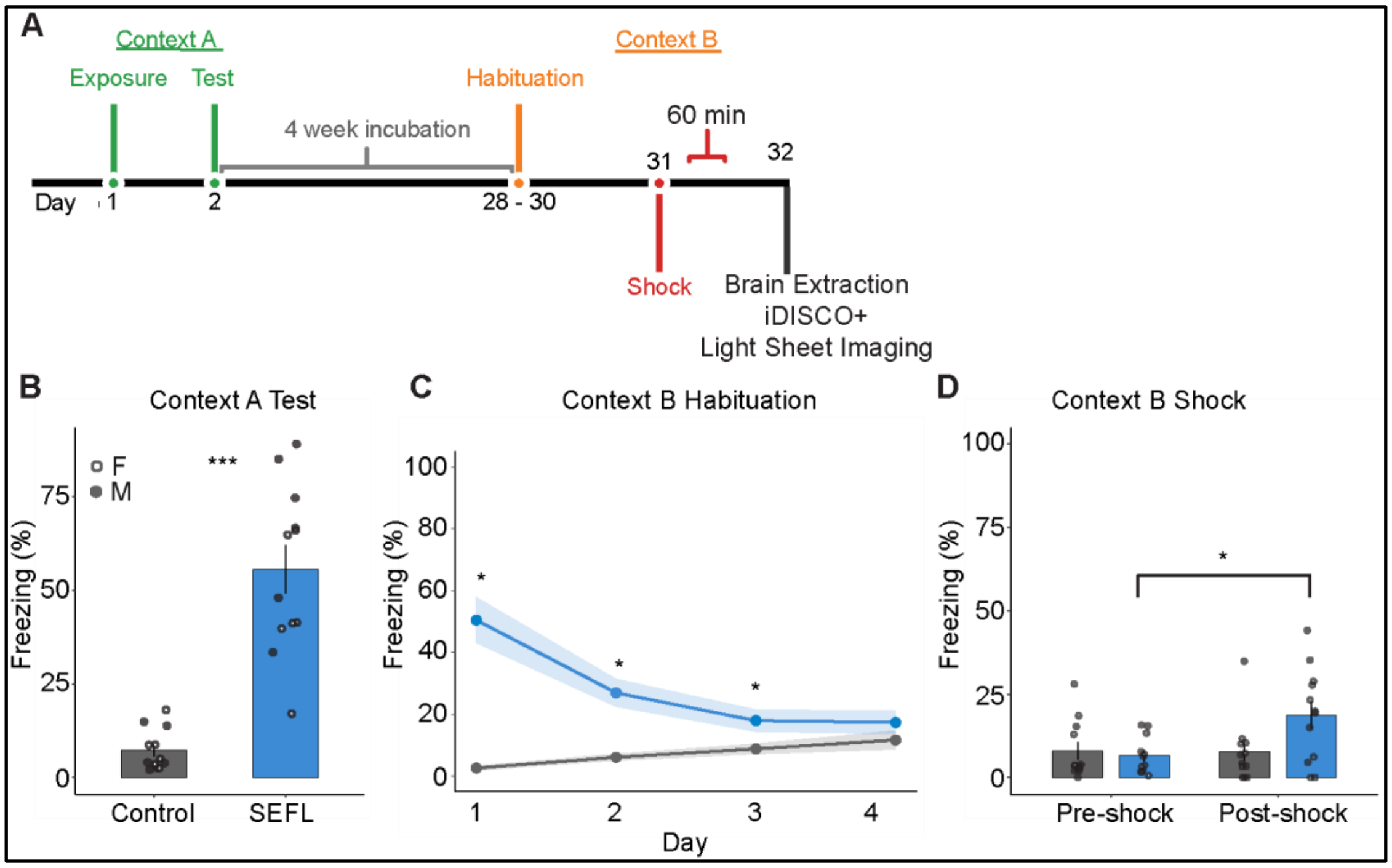

Supplementary Figure 2. Clearing Animals SEFL Behavior (A) Experimental timeline for brain collection following context B fear conditioning. (B) Freezing behavior during Context A Test. (C) Freezing behavior throughout habituation to Context B trials. (D) Freezing behavior during Context B fear acquisition prior to brain collection. * = $p \leq 0.05$, ** = $p \leq 0.01$, *** = $p \leq 0.001$

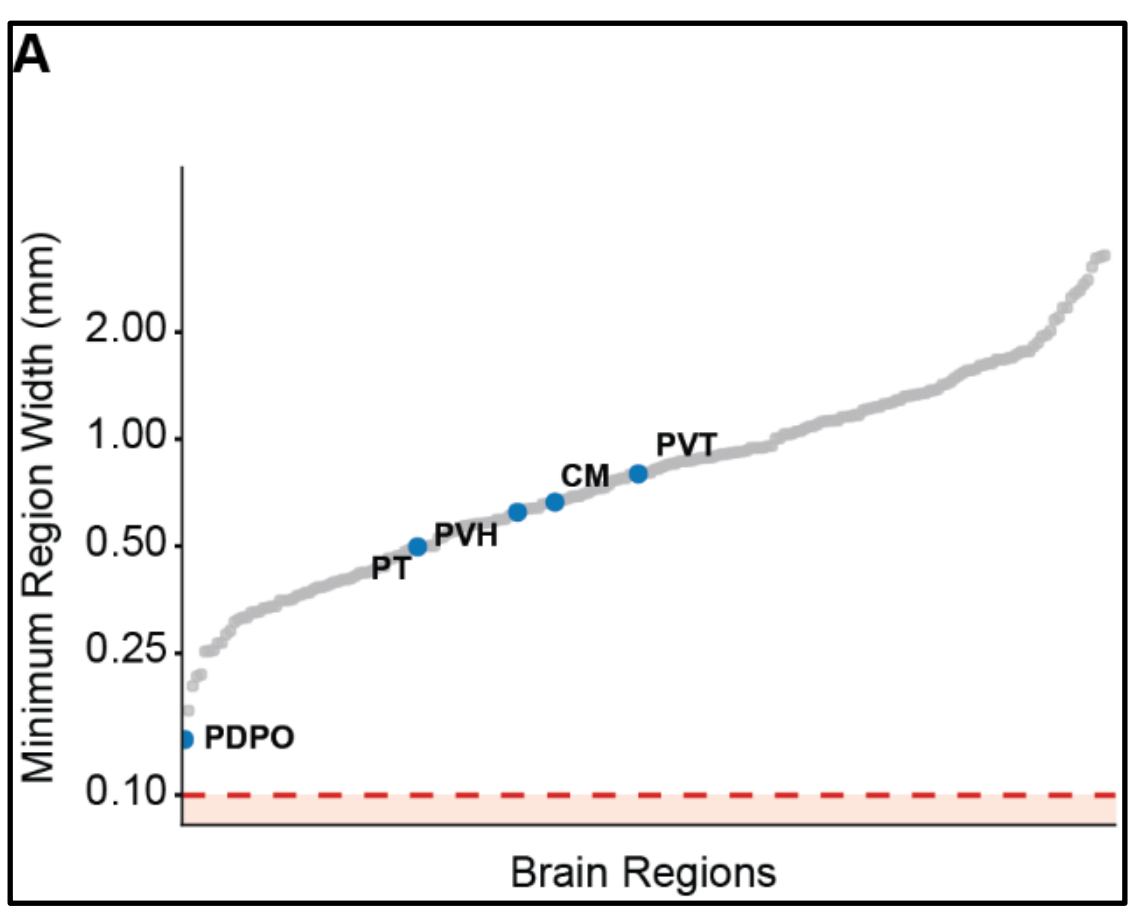

Supplementary Figure 3. Comparison of Minimum Regional Widths Relavtive to Resolution. The imaging and registration pipeline used here has previously been shown to produce registration errors of ~ 0.10 mm at 20 µm voxel resolution (*Reiner et al. 2016; Perens et al. 2021*). We applied this as an exclusion threshold and calculated the minimum effective regional widths of regions included in the analysis. Panel A shows the minimum effective width for each brain region in order of size, demonstrating they are significantly larger than the minimum region size for reliable cell detection and registration.

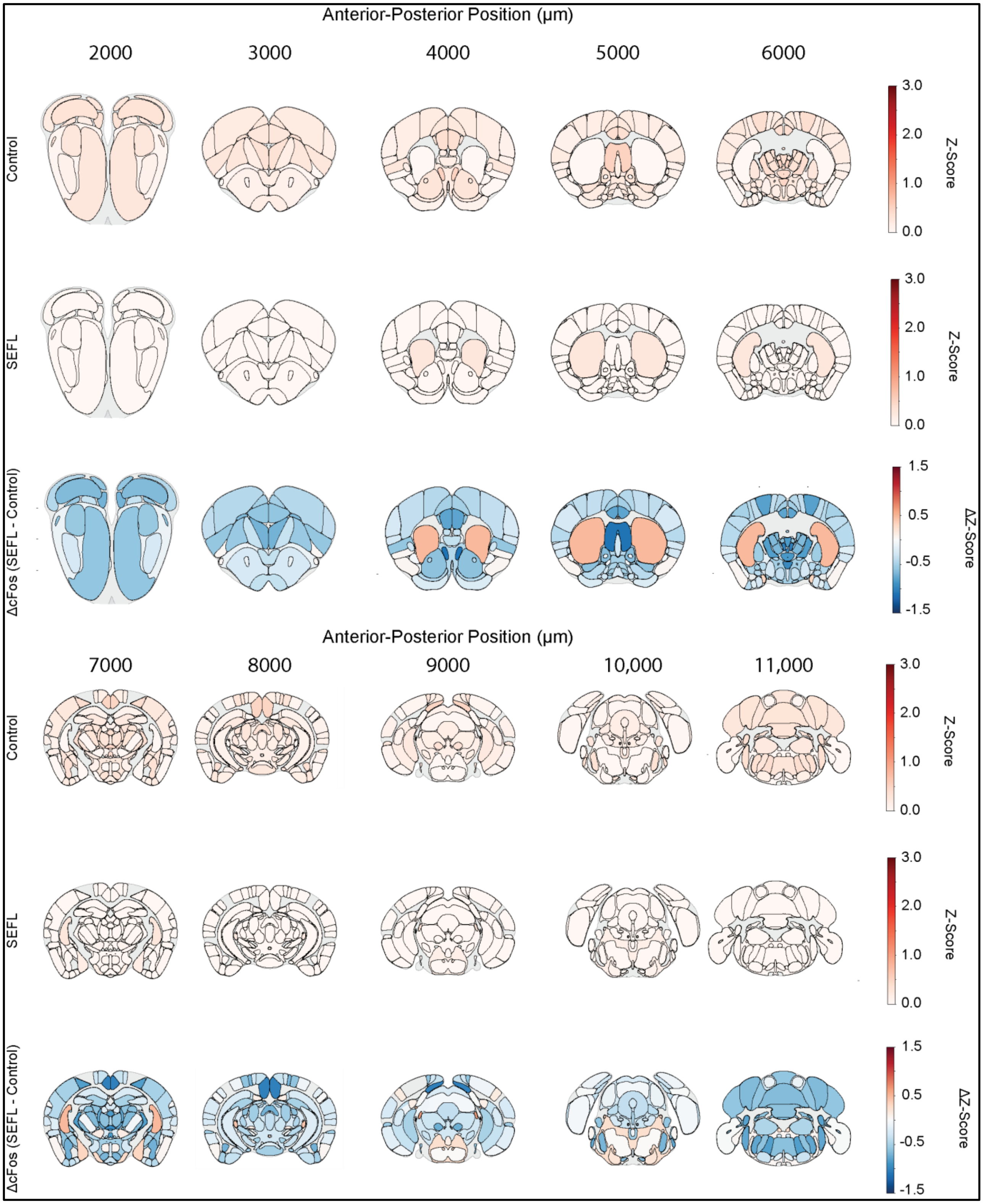

Supplementary Figure 4. Whole-Brain cFos Mapping. Coronal slices showing average z-score cFos density throughout the brain of control (Top) and SEFL (Middle) mice, and average difference heat maps (Bottom). Serial slices at AP = 2,000 um – 11,000 um in 1,000 um steps.

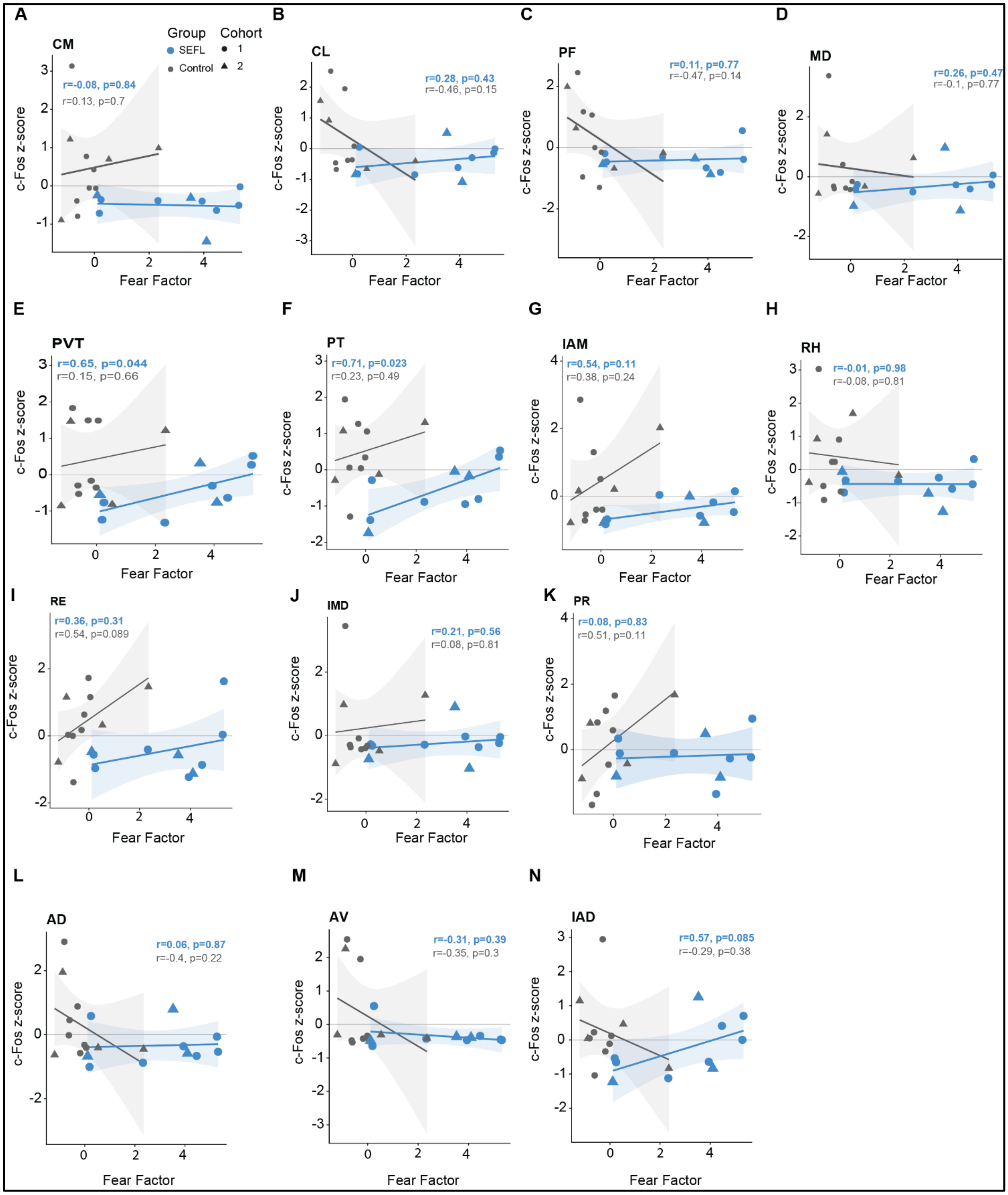

Supplementary Figure 5. Individual Thalamic Nuclei cFos and Sensitization Score Correlations. (A-N) Pearson correlations of normalized cFos expression and sensitization vector (PC1) in control and SEFL mice **(A – D)** Intralaminar Thalamic Nuclei; CM = Central Medial Nucleus of the Thalamus, CL = Central Lateral Nucleus of the Thalamus, PF = Parafascicular

Nucleus, MD = Mediodorsal Nucleus of the Thalamus. **(E-K)** Midline Thalamic Nuclei; PVT = Paraventricular Thalamus, PT = Paratenial Nucleus, IAM = Interanteromedial Nucleus of the Thalamus, RH = , RE = , IMD = Intermediodorsal Nucleus of the Thalamus, PR = Perireunensis Nucleus. **(L-N)** Anterior Thalamic Nuclei; AD = Anterodorsal Nucleus, AV = Anteroventral Nucleus of the Thalamus, IAD = Interanterodorsal Nucleus of the Thalamus.

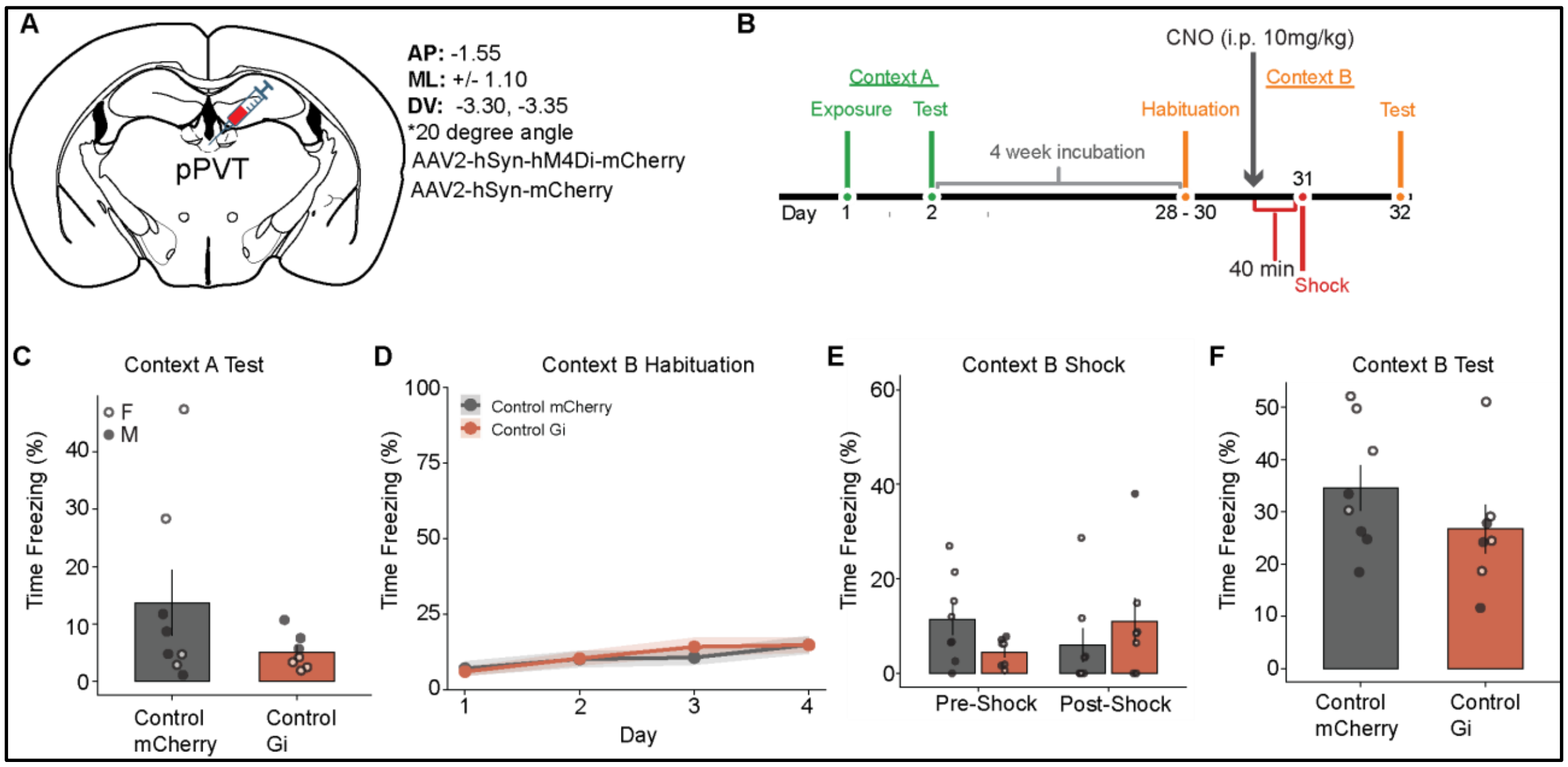

Supplementary Figure 6. Midline Thalamus Inhibition during Context B Fear Acquisition in Control Animals

(A) Viral targeting of midline thalamus with AAV2-HMDi4-mCherry (Control Gi) or AAV2-mCherry (Control mCherry). (B) Experimental schematic for chemogenetic inhibition of midline thalamus during fear acquisition in control animals. (C) Freezing behavior during context A test in control animals. (D) Freezing throughout Context B habituation trials. (E) Freezing behavior during fear acquisition in control mice with chemogenetic inhibition of the midline thalamus. (F) Freezing behavior during Context B fear retrieval test. * = $p \leq 0.05$, ** = $p \leq 0.01$, *** = $p \leq 0.001$

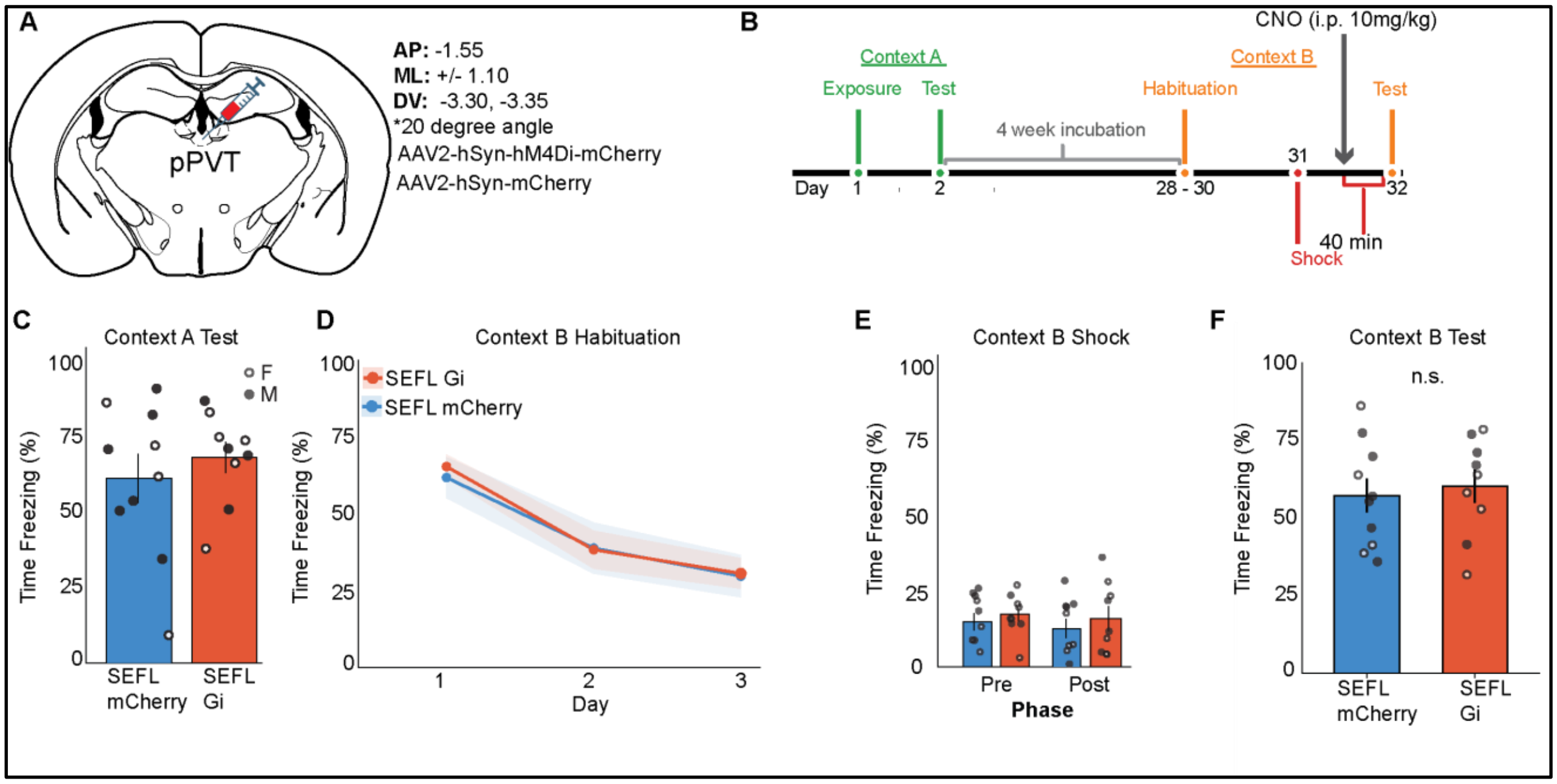

Supplementary Figure 7. Midline Thalamus Inhibition During Context B Fear Test in SEFL Animals
(A) Viral targeting of the midline thalamus with AAV2-HMDi4-mCherry (SEFL Gi) or AAV2-mCherry (SEFL mCherry). (B) Experimental schematic for chemogenetic inhibition of the midline thalamus during fear acquisition in control animals. (C) Freezing behavior during context A test. (D) Freezing throughout Context B habituation trials. (E) Freezing behavior during fear acquisition. (F) Freezing behavior during Context B fear retrieval test with chemogenetic inhibition of the midline thalamus. * = $p \leq 0.05$, ** = $p \leq 0.01$, *** = $p \leq 0.001$

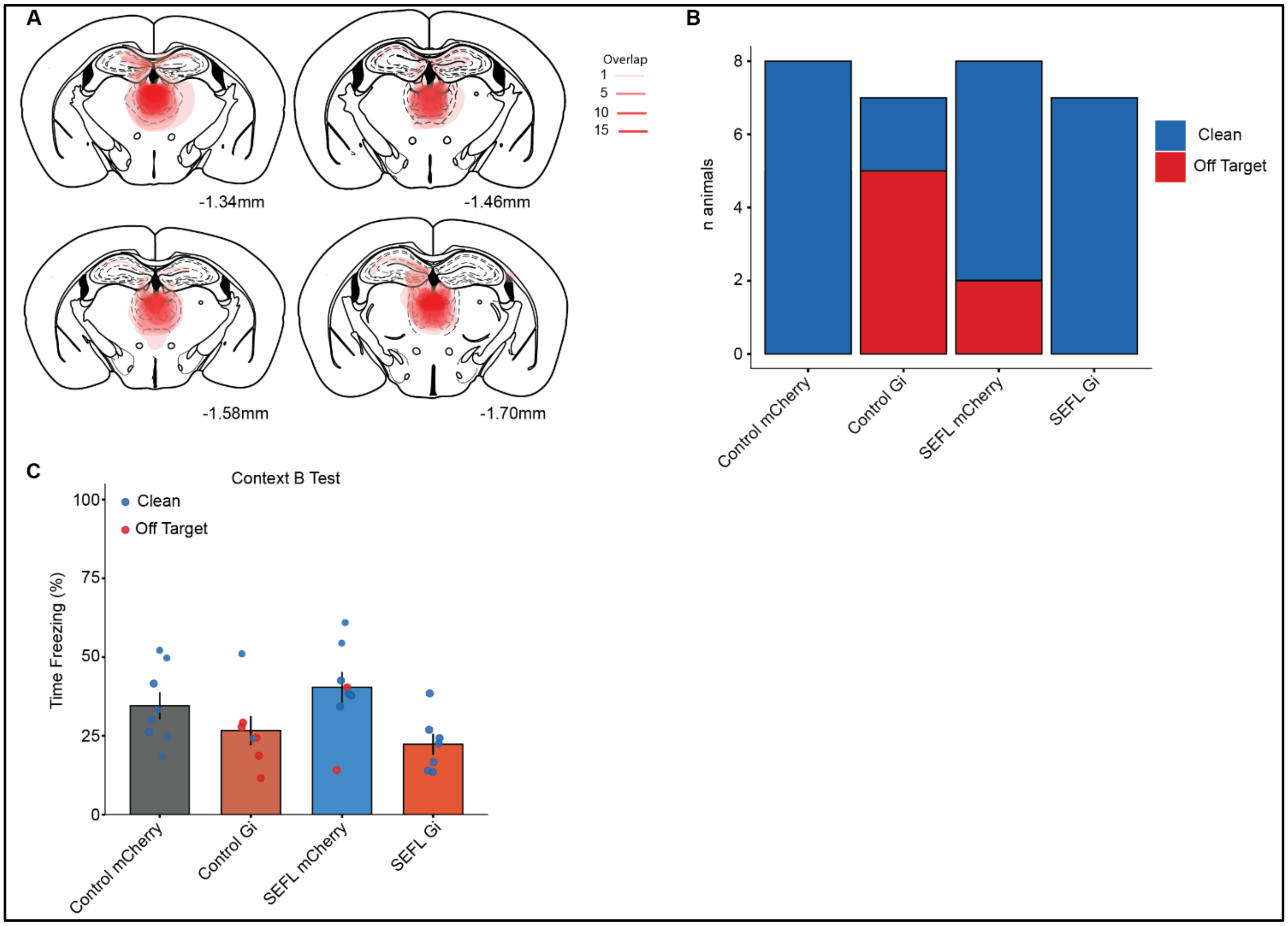

Supplementary Figure 8. Viral Placement Maps & Sensitivity Analysis.
(A) Placement maps showing the extent of bilateral viral expression reconstructed from post-hoc histological verification and plotted onto a shared Allen CCFv3 atlas. Contour lines represent cumulative animal overlap (1, 5, 10, 15 animals) at four coronal sections spanning the PVT (−1.34 to −1.70 mm from bregma). Expression was centered within the PVT across all groups; off-target spread in a subset of animals was largely confined to the injection tract, with sparse cell body labeling in adjacent midline nuclei. (B) Number of animals per experimental group classified as having clean (blue; expression confined to the PVT) or off-target (red; detectable spread to adjacent midline structures) viral expression. All SEFL Gi animals showed clean bilateral PVT expression. Off-target spread was observed in a subset of Control Gi and SEFL mCherry animals. (C) Context B freezing behavior for each group, with individual animals colored by expression category (clean = blue; off-target = red). Within groups containing off-target animals, freezing levels did not differ by expression category, indicating that incidental spread to neighboring midline nuclei did not contribute to the observed behavioral outcomes.
