# Supplementary material for: Whole-brain cFos mapping reveals the paraventricular thalamus as a key node for stress-enhanced fear learning
Source: Front Behav Neurosci. 2026 Sep 8;20:1866792. doi: 10.3389/fnbeh.2026.1866792 (PMC13593807; doi:10.3389/fnbeh.2026.1866792)
Supplement: Supplementary file 2 [file Table_1.pdf]

Supplementary_Table_1_Whole_Brain_All_Regions

Anatomical Abbreviation	Full Region Name	Macroregion / Structure	Atlas Division	Subregion / Layer Depth	Effect Size (LogRR)	Uncorrected P-Value	FDR Corrected P-Value
MARN	Magnocellular reticular nucleus	Hindbrain	Hindbrain	Terminal Node (No further subdivision applied)	-0.176042967	2.29E-28	5.11E-26
SUB	Subiculum	Hippocampal Formation	Hippocampal Formation	Terminal Node (No further subdivision applied)	0.049456772	2.58E-17	2.88E-15
SPF	Subparafascicular nucleus	Thalamus	Thalamus	Terminal Node (No further subdivision applied)	0.072960103	4.57E-05	0.003400403
AT	Anterior group of the dorsal thalamus	Thalamus	Thalamus	Terminal Node (No further subdivision applied)	-1.052948669	1.72E-04	0.009562937
XII	Hypoglossal nucleus	Hindbrain	Hindbrain	Terminal Node (No further subdivision applied)	-1.178864569	0.001337236	0.050289194
CM	Central medial nucleus of the thalamus	Thalamus	Thalamus	Terminal Node (No further subdivision applied)	-0.625821138	0.001353072	0.050289194
AV	Anteroventral nucleus of thalamus	Thalamus	Thalamus	Terminal Node (No further subdivision applied)	-1.313846512	0.003560216	0.101104639
SLD	Sublaterodorsal nucleus	Hindbrain	Hindbrain	Terminal Node (No further subdivision applied)	0.503045043	0.003627072	0.101104639
SF	Septofimbrial nucleus	Cerebral Nuclei	Cerebral Nuclei	Terminal Node (No further subdivision applied)	-0.42895847	0.004297869	0.106423357
PT	Parataenial nucleus	Thalamus	Thalamus	Terminal Node (No further subdivision applied)	-0.328867913	0.004860879	0.106423357
PVH	Paraventricular hypothalamic nucleus	Hypothalamus	Hypothalamus	Terminal Node (No further subdivision applied)	-0.339002651	0.005431209	0.106423357
LSX	Lateral septal complex	Cerebral Nuclei	Cerebral Nuclei	Terminal Node (No further subdivision applied)	-0.305215654	0.005726817	0.106423357
RSPv	Retrosplenial area, ventral part	Cerebral Cortex	Cerebral Cortex	Terminal Node (No further subdivision applied)	-0.961336746	0.007599639	0.127447561
RT	Reticular nucleus of the thalamus	Thalamus	Thalamus	Terminal Node (No further subdivision applied)	-0.537617133	0.008001192	0.127447561
SSp-ll	Primary somatosensory area, lower limb	Cerebral Cortex	Cerebral Cortex	Terminal Node (No further subdivision applied)	-1.286511269	0.010539269	0.143683996
RE	Nucleus of reunions	Thalamus	Thalamus	Terminal Node (No further subdivision applied)	-0.343447834	0.011087843	0.143683996
IAM	Interanteromedial nucleus of the thalamus	Thalamus	Thalamus	Terminal Node (No further subdivision applied)	-0.691148535	0.011220549	0.143683996
IRN	Intermediate reticular nucleus	Hindbrain	Hindbrain	Terminal Node (No further subdivision applied)	-0.397500102	0.011597811	0.143683996
RH	Rhomboid nucleus	Thalamus	Thalamus	Terminal Node (No further subdivision applied)	-0.651418448	0.012621205	0.148133094
CL	Central lateral nucleus of the thalamus	Thalamus	Thalamus	Terminal Node (No further subdivision applied)	-0.530454046	0.015535449	0.167891921
PVT	Paraventricular nucleus of the thalamus	Thalamus	Thalamus	Terminal Node (No further subdivision applied)	-0.320875529	0.01581045	0.167891921
NTB	Nucleus of the trapezoid body	Hindbrain	Hindbrain	Terminal Node (No further subdivision applied)	-0.768875639	0.0192133	0.190988327
PF	Parafascicular nucleus	Thalamus	Thalamus	Terminal Node (No further subdivision applied)	-0.52063974	0.019698348	0.190988327
MD	Mediodorsal nucleus of thalamus	Thalamus	Thalamus	Terminal Node (No further subdivision applied)	-0.532878855	0.022192091	0.197611036
PBG	Parabigeminal nucleus	Midbrain & Pretectum	Midbrain & Pretectum	Terminal Node (No further subdivision applied)	0.804729067	0.023131236	0.197611036
SSp-tr	Primary somatosensory area, trunk	Cerebral Cortex	Cerebral Cortex	Terminal Node (No further subdivision applied)	-1.457849226	0.024186649	0.197611036
SubC	Subceruleus nucleus	Hindbrain	Hindbrain	Terminal Node (No further subdivision applied)	0.63682636	0.024778246	0.197611036
BLA	Basolateral amygdalar nucleus	Cerebral Nuclei	Cerebral Nuclei	Terminal Node (No further subdivision applied)	-0.261715863	0.024812148	0.197611036
GRN	Gigantocellular reticular nucleus	Hindbrain	Hindbrain	Terminal Node (No further subdivision applied)	-0.444055228	0.028224376	0.217035719
FS	Fundus of striatum	Cerebral Nuclei	Cerebral Nuclei	Terminal Node (No further subdivision applied)	-0.343329398	0.029488719	0.219199475
PVHd	Paraventricular hypothalamic nucleus, descending division	Hypothalamus	Hypothalamus	Terminal Node (No further subdivision applied)	-0.206382041	0.035785612	0.257425528
VMH	Ventromedial hypothalamic nucleus	Hypothalamus	Hypothalamus	Terminal Node (No further subdivision applied)	-0.180414413	0.038851347	0.270745321
AI	Agranular insular area	Cerebral Cortex	Cerebral Cortex	Terminal Node (No further subdivision applied)	-0.484106207	0.048515452	0.327846843
VISam	Anteromedial visual area	Cerebral Cortex	Cerebral Cortex	Terminal Node (No further subdivision applied)	-1.544098521	0.051149924	0.335483322
MY-sen	Medulla, sensory related	Hindbrain	Hindbrain	Terminal Node (No further subdivision applied)	-0.479599471	0.054324959	0.346127598
MAM	Mammillary body	Hypothalamus	Hypothalamus	Terminal Node (No further subdivision applied)	-0.345683156	0.056524908	0.350140402
IMD	Intermediodorsal nucleus of the thalamus	Thalamus	Thalamus	Terminal Node (No further subdivision applied)	-0.782739016	0.061941578	0.373225776
ORBl	Orbital area, lateral part	Cerebral Cortex	Cerebral Cortex	Terminal Node (No further subdivision applied)	-0.581757583	0.063599011	0.373225776
CSp	Cortical subplate	Cerebral Cortex	Cerebral Cortex	Terminal Node (No further subdivision applied)	-0.160554232	0.066273996	0.373765735
ACAv	Anterior cingulate area, ventral part	Cerebral Cortex	Cerebral Cortex	Terminal Node (No further subdivision applied)	-0.525367177	0.067724348	0.373765735
GPi	Globus pallidus, internal segment	Cerebral Nuclei	Cerebral Nuclei	Terminal Node (No further subdivision applied)	-0.584328837	0.069374207	0.373765735
ACB	Nucleus accumbens	Cerebral Nuclei	Cerebral Nuclei	Terminal Node (No further subdivision applied)	-0.313293131	0.07039534	0.373765735
BST	Bed nuclei of the stria terminalis	Cerebral Nuclei	Cerebral Nuclei	Terminal Node (No further subdivision applied)	-0.188394488	0.078673518	0.392436201
OPN	Olivary pretectal nucleus	Midbrain & Pretectum	Midbrain & Pretectum	Terminal Node (No further subdivision applied)	-0.514029765	0.078719566	0.392436201
VTA	Ventral tegmental area	Midbrain & Pretectum	Midbrain & Pretectum	Terminal Node (No further subdivision applied)	-0.300780122	0.079191162	0.392436201
AD	Anterodorsal nucleus	Thalamus	Thalamus	Terminal Node (No further subdivision applied)	-0.705644817	0.082430705	0.399585919
PARN	Parvicellular reticular nucleus	Hindbrain	Hindbrain	Terminal Node (No further subdivision applied)	-0.321205277	0.085610114	0.399585919
MOs	Secondary motor area	Cerebral Cortex	Cerebral Cortex	Terminal Node (No further subdivision applied)	-0.691797136	0.086009525	0.399585919
PSV	Principal sensory nucleus of the trigeminal	Hindbrain	Hindbrain	Terminal Node (No further subdivision applied)	-0.430768454	0.093056147	0.409974188
IO	Inferior olivary complex	Hindbrain	Hindbrain	Terminal Node (No further subdivision applied)	-0.69226669	0.093236445	0.409974188
DR	Dorsal nucleus raphe	Midbrain & Pretectum	Midbrain & Pretectum	Terminal Node (No further subdivision applied)	-0.557890221	0.093760913	0.409974188
SSp-bfd	Primary somatosensory area, barrel field	Cerebral Cortex	Cerebral Cortex	Terminal Node (No further subdivision applied)	-0.488332022	0.100811199	0.432324948
LA	Lateral amygdalar nucleus	Cerebral Nuclei	Cerebral Nuclei	Terminal Node (No further subdivision applied)	-0.220146614	0.107247	0.451246809
LP	Lateral posterior nucleus of the thalamus	Thalamus	Thalamus	Terminal Node (No further subdivision applied)	-0.3523192	0.113074577	0.455645623
RN	Red nucleus	Midbrain & Pretectum	Midbrain & Pretectum	Terminal Node (No further subdivision applied)	-0.418532787	0.11450272	0.455645623
PERI	Perirhinal area	Cerebral Cortex	Cerebral Cortex	Terminal Node (No further subdivision applied)	-0.310888185	0.119911919	0.455645623
SI	Substantia innominata	Cerebral Nuclei	Cerebral Nuclei	Terminal Node (No further subdivision applied)	-0.289284213	0.120077457	0.455645623
AAA	Anterior amygdalar area	Cerebral Nuclei	Cerebral Nuclei	Terminal Node (No further subdivision applied)	-0.2197093	0.121097228	0.455645623
MT	Medial terminal nucleus of the accessory optic tract	Midbrain & Pretectum	Midbrain & Pretectum	Terminal Node (No further subdivision applied)	-0.199861011	0.12491131	0.455645623
MOp	Primary motor area	Cerebral Cortex	Cerebral Cortex	Terminal Node (No further subdivision applied)	-0.484733698	0.125620061	0.455645623
PMd	Dorsal premammillary nucleus	Hypothalamus	Hypothalamus	Terminal Node (No further subdivision applied)	-0.394637385	0.128016091	0.455645623
SH	Septohippocampal nucleus	Cerebral Nuclei	Cerebral Nuclei	Terminal Node (No further subdivision applied)	-0.567432755	0.133107894	0.455645623
PMv	Ventral premammillary nucleus	Hypothalamus	Hypothalamus	Terminal Node (No further subdivision applied)	-0.306743895	0.133993168	0.455645623
CP	Caudoputamen	Cerebral Nuclei	Cerebral Nuclei	Terminal Node (No further subdivision applied)	0.366790071	0.135108718	0.455645623
SSp-ul	Primary somatosensory area, upper limb	Cerebral Cortex	Cerebral Cortex	Terminal Node (No further subdivision applied)	-0.585443796	0.136604801	0.455645623
VISpm	posteromedial visual area	Cerebral Cortex	Cerebral Cortex	Terminal Node (No further subdivision applied)	-1.053508676	0.14102307	0.455645623
NPC	Nucleus of the posterior commissure	Midbrain & Pretectum	Midbrain & Pretectum	Terminal Node (No further subdivision applied)	-0.310016113	0.141138263	0.455645623
ORBm	Orbital area, medial part	Cerebral Cortex	Cerebral Cortex	Terminal Node (No further subdivision applied)	-0.286334963	0.142771134	0.455645623
PDPO	Posterodorsal preoptic nucleus	Hypothalamus	Hypothalamus	Terminal Node (No further subdivision applied)	-0.22619171	0.143499319	0.455645623
GU	Gustatory areas	Cerebral Cortex	Cerebral Cortex	Terminal Node (No further subdivision applied)	-0.288099918	0.144197118	0.455645623
SSp-n	Primary somatosensory area, nose	Cerebral Cortex	Cerebral Cortex	Terminal Node (No further subdivision applied)	-0.522092348	0.14542558	0.455645623
GPe	Globus pallidus, external segment	Cerebral Nuclei	Cerebral Nuclei	Terminal Node (No further subdivision applied)	-0.503396897	0.147114282	0.455645623
DG	Dentate gyrus	Hippocampal Formation	Hippocampal Formation	Terminal Node (No further subdivision applied)	-0.18471418	0.152751159	0.466623402
EW	Edinger-Westphal nucleus	Midbrain & Pretectum	Midbrain & Pretectum	Terminal Node (No further subdivision applied)	-0.484192588	0.164353964	0.484885519
MA	Magnocellular nucleus	Cerebral Nuclei	Cerebral Nuclei	Terminal Node (No further subdivision applied)	-0.328440663	0.164379332	0.484885519
IAD	Interanterodorsal nucleus of the thalamus	Thalamus	Thalamus	Terminal Node (No further subdivision applied)	-0.208247719	0.165252464	0.484885519
SS	Somatosensory areas	Cerebral Cortex	Cerebral Cortex	Terminal Node (No further subdivision applied)	-0.263997773	0.168565471	0.487896793
AUDv	Ventral auditory area	Cerebral Cortex	Cerebral Cortex	Terminal Node (No further subdivision applied)	-0.227483383	0.172842362	0.487896793
HY	Hypothalamus	Hypothalamus	Hypothalamus	Terminal Node (No further subdivision applied)	-0.107249711	0.17789944	0.494767296
CLi	Central linear nucleus raphe	Midbrain & Pretectum	Midbrain & Pretectum	Terminal Node (No further subdivision applied)	-0.533090344	0.179713682	0.494767296

**STR**	Striatum	Cerebral Nuclei	Cerebral Nuclei	Terminal Node (No further subdivision applied)	-0.153402855	0.18411962	0.494969095
**ZI**	Zona incerta	Hypothalamus	Hypothalamus	Terminal Node (No further subdivision applied)	-0.17550477	0.184226165	0.494969095
**AUDp**	Primary auditory area	Cerebral Cortex	Cerebral Cortex	Terminal Node (No further subdivision applied)	-0.265779695	0.187945836	0.498951446
**IFR**	Interfascicular nucleus raphe	Midbrain & Pretectum	Midbrain & Pretectum	Terminal Node (No further subdivision applied)	0.227295741	0.192249556	0.504372366
**RCH**	Retrochiasmatic area	Hypothalamus	Hypothalamus	Terminal Node (No further subdivision applied)	-0.179472268	0.199716467	0.517424734
**TRS**	Triangular nucleus of septum	Cerebral Nuclei	Cerebral Nuclei	Terminal Node (No further subdivision applied)	-0.358336587	0.202489414	0.517424734
**RLi**	Rostral linear nucleus raphe	Midbrain & Pretectum	Midbrain & Pretectum	Terminal Node (No further subdivision applied)	-0.458632109	0.204185545	0.517424734
**CLA**	Claustrum	Cerebral Nuclei	Cerebral Nuclei	Terminal Node (No further subdivision applied)	-0.163549899	0.210404008	0.526293135
**MDRN**	Medullary reticular nucleus	Hindbrain	Hindbrain	Terminal Node (No further subdivision applied)	-0.347425798	0.214248706	0.526293135
**ADPO**	Anterodorsal preoptic nucleus	Hypothalamus	Hypothalamus	Terminal Node (No further subdivision applied)	-0.169577105	0.21476536	0.526293135
**CA2**	Field CA2	Hippocampal Formation	Hippocampal Formation	Terminal Node (No further subdivision applied)	-0.246630544	0.218598842	0.529864585
**CS**	Superior central nucleus raphe	Hindbrain	Hindbrain	Terminal Node (No further subdivision applied)	-0.354564345	0.223130604	0.53203002
**TEa**	Temporal association areas	Cerebral Cortex	Cerebral Cortex	Terminal Node (No further subdivision applied)	-0.183364346	0.226336656	0.53203002
**CEA**	Central amygdalar nucleus	Cerebral Nuclei	Cerebral Nuclei	Terminal Node (No further subdivision applied)	-0.121738707	0.22664956	0.53203002
**ORBvl**	Orbital area, ventrolateral part	Cerebral Cortex	Cerebral Cortex	Terminal Node (No further subdivision applied)	-0.318591446	0.22961684	0.533380784
**MPO**	Medial preoptic area	Hypothalamus	Hypothalamus	Terminal Node (No further subdivision applied)	-0.096976044	0.240360746	0.552497779
**VNC**	Vestibular nuclei	Hindbrain	Hindbrain	Terminal Node (No further subdivision applied)	-0.187641881	0.245231	0.552497779
**LPO**	Lateral preoptic area	Hypothalamus	Hypothalamus	Terminal Node (No further subdivision applied)	-0.137201578	0.245279283	0.552497779
**ECTOR**	Ectorhinal area	Cerebral Cortex	Cerebral Cortex	Terminal Node (No further subdivision applied)	-0.194824504	0.251717054	0.56132903
**ACAd**	Anterior cingulate area, dorsal part	Cerebral Cortex	Cerebral Cortex	Terminal Node (No further subdivision applied)	-0.448573364	0.259784779	0.573584215
**MPN**	Medial preoptic nucleus	Hypothalamus	Hypothalamus	Terminal Node (No further subdivision applied)	-0.12401362	0.267886026	0.575802632
**APN**	Anterior pretectal nucleus	Midbrain & Pretectum	Midbrain & Pretectum	Terminal Node (No further subdivision applied)	-0.259139504	0.270934441	0.575802632
**SPZ**	Subparaventricular zone	Hypothalamus	Hypothalamus	Terminal Node (No further subdivision applied)	-0.082228169	0.272290994	0.575802632
**NB**	Nucleus of the brachium of the inferior colliculus	Midbrain & Pretectum	Midbrain & Pretectum	Terminal Node (No further subdivision applied)	0.346801878	0.273329474	0.575802632
**PR**	Perireunensis nucleus	Thalamus	Thalamus	Terminal Node (No further subdivision applied)	-0.198929705	0.276426351	0.575802632
**PSTN**	Parasubthalamic nucleus	Hypothalamus	Hypothalamus	Terminal Node (No further subdivision applied)	0.199324945	0.277356544	0.575802632
**PRN**	Pontine reticular nucleus	Hindbrain	Hindbrain	Terminal Node (No further subdivision applied)	-0.283465303	0.280992803	0.575802632
**AHN**	Anterior hypothalamic nucleus	Hypothalamus	Hypothalamus	Terminal Node (No further subdivision applied)	-0.07339244	0.283046672	0.575802632
**ECU**	External cuneate nucleus	Hindbrain	Hindbrain	Terminal Node (No further subdivision applied)	0.216368714	0.284028204	0.575802632
**PL**	Prelimbic area	Cerebral Cortex	Cerebral Cortex	Terminal Node (No further subdivision applied)	-0.266199966	0.28889537	0.580393401
**AIp**	Agranular insular area, posterior part	Cerebral Cortex	Cerebral Cortex	Terminal Node (No further subdivision applied)	-0.159946873	0.301652527	0.600611728
**SSp-m**	Primary somatosensory area, mouth	Cerebral Cortex	Cerebral Cortex	Terminal Node (No further subdivision applied)	-0.285449313	0.311969036	0.615655708
**SNr**	Substantia nigra, reticular part	Midbrain & Pretectum	Midbrain & Pretectum	Terminal Node (No further subdivision applied)	-0.393662159	0.319683504	0.625345801
**OVLT**	Vascular organ of the lamina terminalis	Hypothalamus	Hypothalamus	Terminal Node (No further subdivision applied)	-0.259139169	0.329310282	0.63857559
**PVp**	Periventricular hypothalamic nucleus, preoptic part	Hypothalamus	Hypothalamus	Terminal Node (No further subdivision applied)	-0.116255672	0.333951199	0.641992391
**NDB**	Diagonal band nucleus	Cerebral Nuclei	Cerebral Nuclei	Terminal Node (No further subdivision applied)	-0.154239946	0.339794963	0.64447616
**CUN**	Cuneiform nucleus	Midbrain & Pretectum	Midbrain & Pretectum	Terminal Node (No further subdivision applied)	-0.209048546	0.34301265	0.64447616
**ARH**	Arcuate hypothalamic nucleus	Hypothalamus	Hypothalamus	Terminal Node (No further subdivision applied)	-0.142725075	0.343913287	0.64447616
**PRE**	Presubiculum	Hippocampal Formation	Hippocampal Formation	Terminal Node (No further subdivision applied)	0.323746066	0.365097033	0.672074878
**AUDpo**	Posterior auditory area	Cerebral Cortex	Cerebral Cortex	Terminal Node (No further subdivision applied)	-0.200369676	0.37070425	0.672074878
**PAG**	Periaqueductal gray	Midbrain & Pretectum	Midbrain & Pretectum	Terminal Node (No further subdivision applied)	-0.171137565	0.370822325	0.672074878
**EP**	Endopiriform nucleus	Cerebral Nuclei	Cerebral Nuclei	Terminal Node (No further subdivision applied)	-0.132639893	0.375125779	0.672074878
**MEPO**	Median preoptic nucleus	Hypothalamus	Hypothalamus	Terminal Node (No further subdivision applied)	-0.206652259	0.375540675	0.672074878
**AIv**	Agranular insular area, ventral part	Cerebral Cortex	Cerebral Cortex	Terminal Node (No further subdivision applied)	-0.1800144	0.376723587	0.672074878
**RSPagl**	Retrosplenial area, lateral agranular part	Cerebral Cortex	Cerebral Cortex	Terminal Node (No further subdivision applied)	-0.369313081	0.394994266	0.694616475
**RSPd**	Retrosplenial area, dorsal part	Cerebral Cortex	Cerebral Cortex	Terminal Node (No further subdivision applied)	-0.286713941	0.395588755	0.694616475
**X**	Nucleus x	Hindbrain	Hindbrain	Terminal Node (No further subdivision applied)	0.167131723	0.406640241	0.708440291
**MG**	Medial geniculate complex	Thalamus	Thalamus	Terminal Node (No further subdivision applied)	-0.279294987	0.412281403	0.708440291
**HPF**	Hippocampal formation	Hippocampal Formation	Hippocampal Formation	Terminal Node (No further subdivision applied)	-0.130140436	0.412992098	0.708440291
**PRM**	Precommissural nucleus	Midbrain & Pretectum	Midbrain & Pretectum	Terminal Node (No further subdivision applied)	-0.151112243	0.426908088	0.726721402
**Y**	Nucleus y	Hindbrain	Hindbrain	Terminal Node (No further subdivision applied)	-0.240282966	0.434446268	0.733950892
**LIN**	Linear nucleus of the medulla	Hindbrain	Hindbrain	Terminal Node (No further subdivision applied)	-0.257696074	0.443695016	0.741887819
**PAS**	Parasolitary nucleus	Hindbrain	Hindbrain	Terminal Node (No further subdivision applied)	0.16420021	0.445798061	0.741887819
**PPN**	Pedunculopontine nucleus	Midbrain & Pretectum	Midbrain & Pretectum	Terminal Node (No further subdivision applied)	-0.13292639	0.457007775	0.753648157
**AVPO**	Anteroventral preoptic nucleus	Hypothalamus	Hypothalamus	Terminal Node (No further subdivision applied)	-0.08622735	0.459623988	0.753648157
**PPSTN**	Preparasubthalamic nucleus	Hypothalamus	Hypothalamus	Terminal Node (No further subdivision applied)	0.13814404	0.46536227	0.757487491
**CA1**	Field CA1	Hippocampal Formation	Hippocampal Formation	Terminal Node (No further subdivision applied)	-0.131116485	0.479278317	0.770656298
**PS**	Parastrial nucleus	Thalamus	Thalamus	Terminal Node (No further subdivision applied)	-0.077242368	0.48036424	0.770656298
**MEA**	Medial amygdalar nucleus	Cerebral Nuclei	Cerebral Nuclei	Terminal Node (No further subdivision applied)	0.058297109	0.484999828	0.772535441
**SSp-ss**	Supplemental somatosensory area	Cerebral Cortex	Cerebral Cortex	Terminal Node (No further subdivision applied)	-0.149950967	0.49312876	0.776816033
**NOT**	Nucleus of the optic tract	Midbrain & Pretectum	Midbrain & Pretectum	Terminal Node (No further subdivision applied)	-0.195870855	0.494654155	0.776816033
**DMX**	Dorsal motor nucleus of the vagus nerve	Hindbrain	Hindbrain	Terminal Node (No further subdivision applied)	-0.198551556	0.49977911	0.779375815
**AUDd**	Dorsal auditory area	Cerebral Cortex	Cerebral Cortex	Terminal Node (No further subdivision applied)	-0.11065286	0.507878908	0.779985061
**VISal**	Anterolateral visual area	Cerebral Cortex	Cerebral Cortex	Terminal Node (No further subdivision applied)	-0.147942762	0.510494219	0.779985061
**PVZ**	Periventricular zone	Hypothalamus	Hypothalamus	Terminal Node (No further subdivision applied)	-0.074018333	0.519226654	0.779985061
**VISC**	Visceral area	Cerebral Cortex	Cerebral Cortex	Terminal Node (No further subdivision applied)	-0.126471798	0.522192827	0.779985061
**VII**	Facial motor nucleus	Hindbrain	Hindbrain	Terminal Node (No further subdivision applied)	-0.171011098	0.527062711	0.779985061
**MB**	Midbrain	Midbrain & Pretectum	Midbrain & Pretectum	Terminal Node (No further subdivision applied)	-0.054416761	0.529244716	0.779985061
**AMB**	Nucleus ambiguus	Hindbrain	Hindbrain	Terminal Node (No further subdivision applied)	-0.217825853	0.530988151	0.779985061
**NLL**	Nucleus of the lateral lemniscus	Hindbrain	Hindbrain	Terminal Node (No further subdivision applied)	0.161172784	0.53618598	0.779985061
**I**	Intercalated amygdalar nucleus	Cerebral Nuclei	Cerebral Nuclei	Terminal Node (No further subdivision applied)	-0.050493601	0.536676605	0.779985061
**PBN**	Parabrachial nucleus	Hindbrain	Hindbrain	Terminal Node (No further subdivision applied)	-0.139588757	0.53818479	0.779985061
**LC**	Locus ceruleus	Hindbrain	Hindbrain	Terminal Node (No further subdivision applied)	-0.218751335	0.538644392	0.779985061
**TH**	Thalamus	Thalamus	Thalamus	Terminal Node (No further subdivision applied)	-0.085673027	0.543263221	0.781598053
**AVPV**	Anteroventral periventricular nucleus	Hypothalamus	Hypothalamus	Terminal Node (No further subdivision applied)	-0.09923954	0.550921799	0.78262255
**TH-sm**	Thalamus, sensory-motor cortex related	Thalamus	Thalamus	Terminal Node (No further subdivision applied)	-0.182381183	0.553342521	0.78262255
**P**	Pons	Hindbrain	Hindbrain	Terminal Node (No further subdivision applied)	0.102056411	0.554603792	0.78262255
**VCN**	Ventral cochlear nucleus	Hindbrain	Hindbrain	Terminal Node (No further subdivision applied)	-0.09492484	0.563795441	0.78262255
**PA**	Posterior amygdalar nucleus	Cerebral Nuclei	Cerebral Nuclei	Terminal Node (No further subdivision applied)	-0.073749757	0.565536907	0.78262255
**MS**	Medial septal nucleus	Cerebral Nuclei	Cerebral Nuclei	Terminal Node (No further subdivision applied)	-0.11634772	0.567028316	0.78262255
**PPT**	Posterior pretectal nucleus	Midbrain & Pretectum	Midbrain & Pretectum	Terminal Node (No further subdivision applied)	-0.143790406	0.568541942	0.78262255
**CA3**	Field CA3	Hippocampal Formation	Hippocampal Formation	Terminal Node (No further subdivision applied)	-0.074202002	0.572435086	0.783147388

IGL	Intergeniculate leaflet of the lateral geniculate complex	Thalamus	Thalamus	Terminal Node (No further subdivision applied)	0.014466502	0.593958571	0.805733134
PGRN	Paragigantocellular reticular nucleus	Hindbrain	Hindbrain	Terminal Node (No further subdivision applied)	-0.117199551	0.596170256	0.805733134
LDT	Laterodorsal tegmental nucleus	Hindbrain	Hindbrain	Terminal Node (No further subdivision applied)	0.145489694	0.601384447	0.807883926
CU	Cuneate nucleus	Hindbrain	Hindbrain	Terminal Node (No further subdivision applied)	-0.113315755	0.612273804	0.813190495
ISOCTX	Isocortex	Cerebral Cortex	Cerebral Cortex	Terminal Node (No further subdivision applied)	-0.082995778	0.614258043	0.813190495
SGN	Suprageniculate nucleus	Thalamus	Thalamus	Terminal Node (No further subdivision applied)	-0.123864508	0.616274411	0.813190495
AP	Area postrema	Hindbrain	Hindbrain	Terminal Node (No further subdivision applied)	0.181849623	0.622749199	0.81690042
ENTl	Entorhinal area, lateral part	Hippocampal Formation	Hippocampal Formation	Terminal Node (No further subdivision applied)	-0.085602644	0.628278468	0.817025529
GR	Gracile nucleus	Hindbrain	Hindbrain	Terminal Node (No further subdivision applied)	-0.181293626	0.633118043	0.817025529
SUM	Supramammillary nucleus	Hypothalamus	Hypothalamus	Terminal Node (No further subdivision applied)	-0.080314609	0.633835949	0.817025529
MY-sat	Medulla, behavioral state related	Hindbrain	Hindbrain	Terminal Node (No further subdivision applied)	-0.159847198	0.642905502	0.820278732
NI	Nucleus incertus	Hindbrain	Hindbrain	Terminal Node (No further subdivision applied)	0.12274118	0.643716494	0.820278732
VP	Ventral posterior complex of the thalamus	Thalamus	Thalamus	Terminal Node (No further subdivision applied)	-0.102203572	0.658605296	0.834482847
PAL	Pallidum	Cerebral Nuclei	Cerebral Nuclei	Terminal Node (No further subdivision applied)	-0.048867665	0.665014712	0.837843395
MEV	Midbrain trigeminal nucleus	Midbrain & Pretectum	Midbrain & Pretectum	Terminal Node (No further subdivision applied)	0.151498557	0.675919008	0.846797409
PHN	Perihypoglossal nuclei	Hindbrain	Hindbrain	Terminal Node (No further subdivision applied)	-0.129933519	0.686141117	0.853241693
TU	Tuberal nucleus	Hypothalamus	Hypothalamus	Terminal Node (No further subdivision applied)	0.054533216	0.691332075	0.853241693
PH	Posterior hypothalamic nucleus	Hypothalamus	Hypothalamus	Terminal Node (No further subdivision applied)	0.060692507	0.701726125	0.853241693
IG	Induseum griseum	Hippocampal Formation	Hippocampal Formation	Terminal Node (No further subdivision applied)	-0.157404838	0.702611723	0.853241693
SCs	Superior colliculus, sensory related	Midbrain & Pretectum	Midbrain & Pretectum	Terminal Node (No further subdivision applied)	-0.128907972	0.704886121	0.853241693
SCN	Suprachiasmatic nucleus	Hypothalamus	Hypothalamus	Terminal Node (No further subdivision applied)	0.04456252	0.70760741	0.853241693
POST	Postsubiculum	Hippocampal Formation	Hippocampal Formation	Terminal Node (No further subdivision applied)	0.156254215	0.707846248	0.853241693
ILA	Infralimbic area	Cerebral Cortex	Cerebral Cortex	Terminal Node (No further subdivision applied)	-0.065833782	0.721887708	0.864996534
MRN	Midbrain reticular nucleus	Midbrain & Pretectum	Midbrain & Pretectum	Terminal Node (No further subdivision applied)	-0.068598741	0.725355838	0.864996534
DCN	Dorsal cochlear nucleus	Hindbrain	Hindbrain	Terminal Node (No further subdivision applied)	0.057713712	0.749913579	0.88952515
VISpl	Posterolateral visual area	Cerebral Cortex	Cerebral Cortex	Terminal Node (No further subdivision applied)	-0.090156533	0.754960191	0.890773136
FF	Fields of Forel	Hypothalamus	Hypothalamus	Terminal Node (No further subdivision applied)	-0.092291477	0.767994391	0.90138289
BA	Bed nucleus of the accessory olfactory tract	Cerebral Nuclei	Cerebral Nuclei	Terminal Node (No further subdivision applied)	0.041877884	0.774872368	0.904693917
LT	Lateral terminal nucleus of the accessory optic tract	Midbrain & Pretectum	Midbrain & Pretectum	Terminal Node (No further subdivision applied)	0.065348398	0.77956383	0.905430907
VISp	Primary visual area	Cerebral Cortex	Cerebral Cortex	Terminal Node (No further subdivision applied)	-0.08465884	0.787782675	0.91005446
STN	Subthalamic nucleus	Hypothalamus	Hypothalamus	Terminal Node (No further subdivision applied)	-0.094784746	0.794755955	0.91005446
LHA	Lateral hypothalamic area	Hypothalamus	Hypothalamus	Terminal Node (No further subdivision applied)	-0.026119745	0.795787533	0.91005446
ENTm	Entorhinal area, medial part, dorsal zone	Hippocampal Formation	Hippocampal Formation	Terminal Node (No further subdivision applied)	-0.045111184	0.816953536	0.929493053
VM	Ventral medial nucleus of the thalamus	Thalamus	Thalamus	Terminal Node (No further subdivision applied)	0.035629467	0.826823848	0.935947808
IPN	Interpeduncular nucleus	Midbrain & Pretectum	Midbrain & Pretectum	Terminal Node (No further subdivision applied)	0.057364323	0.835346819	0.940440242
PP	Peripeduncular nucleus	Thalamus	Thalamus	Terminal Node (No further subdivision applied)	0.049333021	0.839872481	0.940440242
SO	Supraoptic nucleus	Hypothalamus	Hypothalamus	Terminal Node (No further subdivision applied)	-0.024158201	0.847484692	0.940440242
RPO	Nucleus raphe pontis	Hindbrain	Hindbrain	Terminal Node (No further subdivision applied)	-0.072141755	0.848149582	0.940440242
VLPO	Ventrolateral preoptic nucleus	Hypothalamus	Hypothalamus	Terminal Node (No further subdivision applied)	-0.022232671	0.858087095	0.940440242
P-mot	Pons, motor related	Hindbrain	Hindbrain	Terminal Node (No further subdivision applied)	0.034408092	0.859580369	0.940440242
SOC	Superior olivary complex	Hindbrain	Hindbrain	Terminal Node (No further subdivision applied)	0.034278432	0.860609929	0.940440242
IC	Inferior colliculus	Midbrain & Pretectum	Midbrain & Pretectum	Terminal Node (No further subdivision applied)	-0.046501339	0.864530267	0.940440242
PPy	Parapyramidal nucleus	Hindbrain	Hindbrain	Terminal Node (No further subdivision applied)	0.049051577	0.878927566	0.945833287
DMH	Dorsomedial nucleus of the hypothalamus	Hypothalamus	Hypothalamus	Terminal Node (No further subdivision applied)	0.023090589	0.879295857	0.945833287
MPT	Medial pretectal area	Midbrain & Pretectum	Midbrain & Pretectum	Terminal Node (No further subdivision applied)	-0.045417229	0.882212214	0.945833287
ME	Median eminence	Hypothalamus	Hypothalamus	Terminal Node (No further subdivision applied)	-0.03122074	0.905118398	0.965748338
POL	Posterior limiting nucleus of the thalamus	Thalamus	Thalamus	Terminal Node (No further subdivision applied)	0.027974924	0.931938942	0.973480571
MRN-r	Midbrain reticular nucleus, retrorubral area	Midbrain & Pretectum	Midbrain & Pretectum	Terminal Node (No further subdivision applied)	0.029198344	0.932870298	0.973480571
SAG	Nucleus sagulum	Midbrain & Pretectum	Midbrain & Pretectum	Terminal Node (No further subdivision applied)	0.024232662	0.933381121	0.973480571
MY	Medulla	Hindbrain	Hindbrain	Terminal Node (No further subdivision applied)	0.011401965	0.935275674	0.973480571
LH	Lateral habenula	Thalamus	Thalamus	Terminal Node (No further subdivision applied)	-0.020631567	0.938228947	0.973480571
TM	Tuberomammillary nucleus	Hypothalamus	Hypothalamus	Terminal Node (No further subdivision applied)	-0.008805316	0.943465561	0.973480571
NTS	Nucleus of the solitary tract	Hindbrain	Hindbrain	Terminal Node (No further subdivision applied)	-0.013221198	0.950501936	0.973480571
MH	Medial habenula	Thalamus	Thalamus	Terminal Node (No further subdivision applied)	-0.021014228	0.95586048	0.973480571
PAR	Parasubiculum	Hippocampal Formation	Hippocampal Formation	Terminal Node (No further subdivision applied)	-0.016765353	0.955960319	0.973480571
SCm	Superior colliculus, motor related	Midbrain & Pretectum	Midbrain & Pretectum	Terminal Node (No further subdivision applied)	-0.01253782	0.956019036	0.973480571
VISl	Lateral visual area	Cerebral Cortex	Cerebral Cortex	Terminal Node (No further subdivision applied)	0.011848771	0.962748727	0.975877119
PVP	Periventricular hypothalamic nucleus, posterior part	Hypothalamus	Hypothalamus	Terminal Node (No further subdivision applied)	-0.008927416	0.968317597	0.977080652
KF	Kolliker-Fuse subnucleus	Hindbrain	Hindbrain	Terminal Node (No further subdivision applied)	0.00297311	0.990825598	0.995288776
SNc	Substantia nigra, compact part	Midbrain & Pretectum	Midbrain & Pretectum	Terminal Node (No further subdivision applied)	-0.002145866	0.995463486	0.995463486
